# Safe production of *Aspergillus terreus* xylanase from *Ricinus communis*: gene identification, molecular docking, characterization, production of xylooligosaccharides, and its biological activities

**DOI:** 10.1186/s43141-022-00390-9

**Published:** 2022-08-12

**Authors:** Shaimaa A. Nour, Ghada M. El-Sayed, Hanan A. A. Taie, Maha T. H. Emam, Ahmed F. El-Sayed, Rasha G. Salim

**Affiliations:** 1 Chemistry of Natural and Microbial Products Department, Pharmaceutical and Drug Industries Research Institute, National Research Centre, 33 El-Bohouth St. (Former El-Tahrir St.), Dokki, Cairo, 12622 Egypt; 2 Microbial Genetic Department, Biotechnology Research Institute, National Research Centre, 33 El-Bohouth St. (Former El-Tahrir St.), Dokki, Cairo, 12622 Egypt; 3 Plant Biochemistry Department, Agricultural and Biology Research Institute, National Research Centre, 33 El-Bohouth St. (Former El-Tahrir St.), Dokki, Cairo, 12622 Egypt; 4 Genetics & Cytology Department, Biotechnology Research Institute, National Research Centre, 33 El-Bohouth St. (Former El-Tahrir St.), Dokki, Cairo, 12622 Egypt

**Keywords:** *Aspergillus terreus*, Xylanase, Optimization, Characterization, Molecular identification, Gene isolation, Molecular docking

## Abstract

**Background:**

The production of industrial enzymes such as xylanase using sufficient cost-effective substrates from potent microorganisms is considered economically feasible. Studies have reported castor cake (*Ricinus communis*) as the most potent and inexpensive alternative carbon source for production of xylanase C by using *Aspergillus terreus* (*A. terreus*).

**Results:**

*A. terreus* strain RGS Eg-NRC, a local isolate from agro-wastes, was first identified by sequencing the internal transcribed spacer region of a nuclear DNA encoding gene cluster deposited in GenBank (accession number MW282328). Before optimization of xylanase production, *A. terreus* produced 20.23 U/g of xylanase after 7 days using castor cake as a substrate in a solid-state fermentation (SSF) system that was employed to achieve ricin detoxification and stimulate xylanase production. Physicochemical parameters for the production of xylanase were optimized by using a one-variable-at-a-time approach and two statistical methods (two-level Plackett–Burman design and central composite design, CCD). The maximum xylanase yield after optimization was increased by 12.1-fold (245 U/g). A 60–70% saturation of ammonium sulfate resulted in partially purified xylanase with a specific activity of 3.9 IU/mg protein. At 60 °C and pH 6, the partially purified xylanase had the highest activity, and the activation energy (Ea) was 23.919 kJmol. Subsequently, antioxidant capacity and cytotoxicity tests in normal Ehrlich ascites carcinoma human cells demonstrated xylooligosaccharides produced by the xylanase degradation of xylan as a potent antioxidant and moderate antitumor agent. Further investigations with sodium dodecyl sulfate polyacrylamide gel electrophoresis then determined the molecular weight of partially purified xylanase C to be 36 kDa. Based on the conserved regions, observations revealed that xylanase C belonged to the glycosyl hydrolase family 10. Next, the xylanase-encoding gene (xynC), which has an open reading frame of 981 bp and encodes a protein with 326 amino acids, was isolated, sequenced, and submitted to the NCBI GenBank database (accession number LC595779.1). Molecular docking analysis finally revealed that Glu156, Glu262, and Lys75 residues were involved in the substrate-binding and protein-ligand interaction site of modeled xylanase, with a binding affinity of −8.7 kcal. mol^−1.^

**Conclusion:**

The high production of safe and efficient xylanase could be achieved using economical materials such *as Ricinus communis*.

## Background

Xylan is the major hemicellulosic component of agricultural residues. It is a polysaccharide composed of a xylose backbone unit chain linked by β-1,4bonds and branched with saccharide and phenolic side chains at various positions [1]. Alternatively, xylanolytic enzymes, such as endo β-1,4-xylanase, are industrially important enzymes that hydrolyze xylan backbones by cleaving 1,4-glycosidic linkages, forming xylose, xylobiose, xylotriose, and xylooligosaccharides (XOS) of various lengths [2]. Therefore, xylanases have recently attracted considerable attention because of their biotechnological potential in a several industrial applications such as food industry, animal feed, textile industry, paper and pulp industry, and the generation of biofuels when combined with several enzymes such as ethanol and xylitol from lignocellulosic residues [3]. Furthermore, studies have reported that although the main microbial sources in xylanases are fungi, bacteria, and actinomycetes [4], filamentous fungi are particularly interesting because they secrete enzymes into the medium in higher quantities than those found in yeasts and bacteria [5]. Moreover, although the most industrial xylanase-producing fungi are the *Trichoderma* and *Aspergillus* species [6], *Aspergillus* spp. are considered suitable host organisms for the production of industrial enzymes due to their high secretion ability, rapid growth on inexpensive media, and they are generally regarded as safe (GRAS) [7].

The castor bean (*Ricinus communis*) is a species of the Euphorbiaceae family, which flowers in tropical and subtropical climates worldwide [8]. Since the presence of poisonous components in castor seeds has remained a key stumbling block in the consumption of castor seeds, so various detoxification methods have been tried, with varying degrees of benefit and problems [9]. Fermentation is an ancient biotechnology method in which microbes are used to manufacture secondary metabolites and recombinant products on a large scale [10].

Hence, to help with the production process of traditional meals and minimize toxins in various substrates, microorganisms (fungi), for example, *Aspergillus terreus* (*A. terreus*), have been implemented However, although some strains of *A. terreus* generate mycotoxins, which are toxic to both animals and humans [11], a preliminary assessment of mycotoxin production in *Aspergillus* was conducted. Their results showed nonexistent mycotoxin production [12]. Also, fermentation methods can improve food safety by lowering harmful chemicals during manufacturing [13].

Since the cost of enzymes is one of the most important considerations in determining the overall economics of a process, efficient industrial applications reduce the costs of enzyme synthesis by improving the fermentation medium. More optimal designs for high enzyme production are necessary. Notable methods are statistical approaches, such as response surface methodology (RSM), which can achieve high enzyme production in minimal experiments. Therefore, RSM has recently been used successfully to improve product output while reducing the time and expense of biotechnological processes [14]. Research on discovering novel xylanases is still ongoing [15]. According to the amino acid sequence similarity, while xylanases are classified into glycosyl hydrolase (GH) families 10 (GH10) and 11 (GH11), more GH families such as 5, 8, and 43 have been discovered using the CAZy database. In contrast, xylanases with large molecular mass (30 kDa), acidic isoelectric point (pI) values, and a (b/a) 8-barrel configuration have been found in GH 10. Previously, Henrissat and Bairoch classified GH 11 as low-molecular-weight endoxylanase, classified into alkaline and acidic pI types [16]. However, various enzymes from the same family do not always have the same enzymatic characteristics [17]. For instance, while most endo-1, xylanase family 10 have a high molecular weight (> 30 kDa) and a low pI, xylanases with a high pI and a low molecular weight (< 30 kDa) cover family 11 [11]. Based on these inconsistencies and facts, this study investigated the sequence, characterization, and molecular docking of the xylanase gene (xynC) from *A. terreus* understand substrate recognition of this thermostable xylanase. Subsequently, we provided a basis for the rational design of the interaction between xylanase and xylan, making this study the first to report the molecular docking of xylanase produced by *A. terreus*.

This study first achieved the detoxification of castor seeds through solid-state fermentation for xylanase production from *A. terreus*. Then, the enhancement of xylanase enzyme production by RSM was conducted, after which the gene-encoded xylanase was isolated and sequenced. Subsequently, the enzyme was biochemically characterized, and its enzyme-ligand interaction was studied. Xylooligosaccharide production was implemented, and its antioxidant and antitumor activities were finally evaluated.

## Methods

### Isolation of xylan-degrading fungus

A genus of *Aspergillus* was isolated and characterized morphologically from the natural composting of agro-wastes and kept in the fungal culture collection of the Microbial Genetics Laboratory, National Research Centre, Egypt, and maintained in PDA medium consists of 2% potato broth, 2% dextrose, and 2% agar (Merck, USA).

### Enzyme production

The following steps were followed to assess the production of xylanase from *Aspergillus*: Five grams of preliminarily autoclaved dry weights of eight different agricultural wastes (garlic peels, onion peels, palm kernel wastes, pomegranate peels, potato peels, wheat brane and *Ricinus communis*, sesame waste from an oil mill at the Egypt’s National Research Center) was used to produce enzymes in a 250 mL Erlenmeyer flask wet with 10 mL tap water. After scraping the cultivated slant with 10 mL sterilized distilled water containing 0.1% tween 80, the pre-culture was injected into the previously autoclaved media and incubated for 7 days at 30 °C.

After incubation on an orbital shaker (150 rpm) for 1 h, the fermented substrate was extracted with 50 mL distilled water and centrifuged at 5000 rpm at 4 °C for 10 min. The enzyme analysis was performed on the generated supernatant [18].

### Enzyme and protein estimation

Xylanase activity was assayed according to [19] by incubating 0.5 mL of diluted enzyme extract and 0.5 mL of 1% (w/v) xylan from beech wood (Sigma, USA) solution in 50 mM sodium phosphate buffer pH 5.8 at 50 °C for 30 min; the enzyme reaction was stopped by adding 3.0 mL of DNSA (3,5-dinitrosalicylic acid was obtained from PanReac, Barcelona, Spain) reagent and boiling for 5 min. After cooling, the xylose sugar emitted was detected at 540 nm after cooling. One unit of xylanase activity (U) is defined as the quantity of xylanase that releases 1 mol of xylose per mL of crude enzyme extract per minute. Protein content was determined according to [20]. Bovine serum albumin was used as a standard.

### Cytotoxic effect on human normal fibroblast cell line (BJ1)

Cytotoxic effect of the xylanase was determined using MTT (3-(4, 5-dimethylthiazol-2-yl)-2, 5-diphenyl tetrazolium bromide) to purple formazan [21, 22]. The viability was calculated according to the formula (reading of extract/reading of negative control) (−1) × 100. A probit analysis was carried for IC50 and IC90 determination using SPSS 11 program.

### Molecular identification of xylanase producing Aspergillus

The fungal isolate, *Aspergillus*, was detected based on its morphology under the light microscope (Olympus CH40RF200, Japan). Here, the genus was exposed to molecular identification for identification to species level. The genomic DNA was extracted using GeneJET Genomic DNA purification kit cat. no. K0721 (Thermo Scientific, Lithuania) according to manufacturer’s instruction manual. Polymerase chain reaction (PCR) was achieved to amplify sequencing of the fragment of the nuclear ribosomal gene cluster containing internal transcribed spacers and 5.8S rRNA gene using the following primers: ITS1 (5′CTTGGTCATTTAGAGGAAGTAA3′), and ITS4 (5′GCTGCGTTCTTCATCGATGC3′) [23]. The PCR cycles consisted of an initial denaturation step for 5 min at 94 °C followed by 35 cycles of denaturation for 30 s at 94 °C, annealing for 30 s at 55 °C, and amplification for 1 min at 72 °C, with a final extension step for 5 min at 72 °C. PCR product was analyzed on a 1% agarose gel by electrophoresis using a 100-bp ladder DNA marker (Invitrogen, California, USA). The gel was visualized and photographed using ™XR + Gel Documentation System (Bio-Rad, California 94547, USA). Purified PCR product was subjected to sequencing by Sanger sequencing method using sequencer 3500 genetic analyzer, big dye X terminator kit (Thermo Fisher, USA) for forward and reverse directions in biomedical laboratory of colors (Clinilab, Egypt). BLASTn (https://www.ncbi.nlm.nih.gov/blast/Blast.cgi) was used to detect the evolutional relationship with other relatives. Multiple sequence alignment of ITS sequence from this study plus ITS retrieved sequences from GenBank was performed using MUSCLE algorithm [24] available in MEGA X [25]. The evolutionary history was inferred using the neighbor-joining method [26].

### Optimization of cultural conditions for xylanase production

#### One variable at a time (OVAT)

To increase the enzyme productivity from the fungal isolate *A. terreus* RGS Eg-NRC, the enzyme production conditions were improved one-variable-at-a-time replace 10 ml tap water with four mineral salt solutions, MAI: MgSO_4_.7H_2_O, 0.5; KH_2_PO_4_, 1.5; and pH 7.0; MAII: MgSO_4_.7H_2_O, 0.5; KH_2_PO_4_, 1.5; yeast extract, 2; peptone, 2; and pH 7.0. MAIII: Na_2_HPO_4_, 11; NaH_2_PO_4_, 6; KCl, 3; MgSO_4_.7H_2_O, 0.1; and pH 7.0; MAIV: KH_2_PO_4_, 0.1; (NH_4_)H_2_PO_4_, 1; MgSO_4_.7H_2_O, 0.5; CaCl_2_.2H_2_O, 1; FeSO_4_, 10; MnSO_4_, 10; and pH 7.0, and were prepared in distilled water as [27]. Besides these salt solutions, one moistening agent was distilled water. After that, the most suitable concentration of the selected nitrogen source was investigated in the range of 0.1–4% after the addition of nitrogen sources with equivalent value to the media. Then, there are the mono sugar additions to consider.

### Statistical optimization

Two-step optimization approaches were carried out to enhance xylanase production.

#### Plackett and Burman (PB)

Seven different factors that have the highest influence on the enzyme production were chosen to perform this optimization [28] including the following: *Ricinus communis* cake as a carbon source, corn steep liquor (CSL) as a nitrogen source, KH_2_PO_4_, glucose, moistening agent, inoculum size, and incubation period.

As indicated in Table 2, the variables were represented at two levels: high concentration (+1) and low concentration (−1) in eleven trials. Each row represents a trial run, and each column reflects the concentration of an independent variable. The first-order linear model is used in the Plackett–Burman experimental design:

1 $$Y={\mathrm{B}}_0+\Sigma\ {\mathrm{B}}_{\mathrm{i}}\ {\mathrm{X}}_{\mathrm{i}}$$

where *Y* is the response (XY biosynthesis), B0 is the model intercept, and *B*i is the variables’ estimates. The effect of each variable was determined by the following equation:

2 $$E\ \left({\mathrm{X}}_{\mathrm{i}}\right)=2\left({\Sigma \mathrm{M}}_{\mathrm{i}+}-{\Sigma \mathrm{M}}_{\mathrm{i}-}\right)/\mathrm{N}$$

Mi+ and Mi reflect XY production from trials in which the variable (Xi) measured was present at high and low concentrations, respectively, and N represents the number of trials.

The square root of each effect’s variance was used to calculate the standard error (SE), and the significance level (*p*-value) of each concentration effect was assessed using the student’s *t*-test:

3 $$t\ \left({\mathrm{X}}_{\mathrm{i}}\right)=\mathrm{E}\left({\mathrm{X}}_{\mathrm{i}}\right)/\mathrm{SE}$$

where *E* (X_i_) is the effect of variable *X*_i_.

#### Central composite design

Three variables (carbon, moistening agent, and time) were selected for RSM of CCD. The independent variables were evaluated at five distinct levels in the experimental design; the second-order polynomial function was used to correlate the relationship between the independent variables and the predicted response Y, Eq. (4).

4 $${Y}_{\mathrm{Activity}}={\upbeta}_0+{\upbeta}_1{\mathrm{X}}_1+{\upbeta}_2{\mathrm{X}}_2+{\upbeta}_3{\mathrm{X}}_3+{\upbeta}_{11}{\mathrm{X}}_{12}+{\upbeta}_{22}{\mathrm{X}}_{22}+{\upbeta}_{33}\ {\mathrm{X}}_{32}+{\upbeta}_{12}{\mathrm{X}}_1{\mathrm{X}}_2+{\upbeta}_{13}{\mathrm{X}}_1{\mathrm{X}}_3+{\upbeta}_{23}{\mathrm{X}}_2{\mathrm{X}}_3$$

*Y*_Activity_ is the predicted xylanase production (U/mL), β_0_ was the model constant, *X*_1_, *X*_2_, and *X*_3_ (*Ricinus communis* cake, moistening agent and time respectively) were the independent variables, the linear coefficients were β_1_, β_2_, and β_3_, the cross-product coefficients were *β*_12_, *β*_13_, and *β*_23_, and the quadratic coefficients were β_11_, β_22_, and β_33_, respectively. The regression analysis of the experimental data was performed using “SPSS” Version 15.0. The *R*^2^ coefficient of determination was used to represent the fit quality of the polynomial model equation. The experiments were repeated three times, with the mean values reported.

### Xylanase partial purification through sodium dodecyl sulfate-polyacrylamide gel electrophoresis

Fractional precipitation with ethanol and acetone, including salting out with ammonium sulfate at 30–90% concentrations and 10% intervals, was used to partially purify the crude enzyme. Each fraction’s enzyme activity and protein content were then measured [29], after which molecular weight and purity were determined by SDS-PAGE according to a previous study [30]. The protein sample and sample application buffer comprised 4% SDS, 20% glycerol, 200 mM DTT, 0.01% bromophenol blue, and 0.1 M Tris HCl (pH 6.8), taken in the ratio 1:1 and mixed well. Then, this mixture was heated at 100 °C for 5 min and centrifuged at 1000 rpm for 10 min at 4 °C. Thus, the sample was ready to be loaded.

First, the gel was washed with distilled water. Then, Coomassie brilliant blue R–250 staining was used to visualize the proteins in the gel [31], after which native PAGE was used to conduct zymo-gram investigations. Next, samples of the ammonium sulfate-precipitated fraction and partially purified enzyme produced by ion-exchange chromatography were placed in appropriate wells after casting and solidifying the polyacrylamide gel. Subsequently, the polyacrylamide gel was over layered onto an agar gel after electrophoresis at 200 V for 1 h in a Tris-glycine buffer (pH 8.3), after which the polyacrylamide gel was over layered onto an agar gel (1.5%, w/v) preceded with 0.5% xylan and incubated for another hour at 45 °C. Finally, the xylanase site was determined by staining the agar gel with 0.1% Congo red (prestained protein ladder (10–250 kDa, Thermo Scientific) and was used as a molecular weight marker] [32].

### Biochemical characterization of Xylanase C

#### Influence of pH on the activity and stability of enzymes

Phosphate buffer solutions determined the *A. terreus* xylanase activity at different pH conditions (4.0–9.0). Then, without a substrate, the pH stability of the enzyme was tested by incubating it at room temperature for various durations in the abovementioned buffer at pH ranging from 4 to 8. Finally, the residual enzyme activity was calculated under normal assay conditions.

#### Temperature effects on enzyme activity and stability

The effect of temperature on xylanase activity was examined by operating the reaction at different temperatures, ranging from 40 to 60 °C for 30 min. First, the temperature with the highest enzyme activity was determined to be the optimum temperature for the enzyme. Subsequently, the remaining xylanase activity (percent) at each temperature was calculated, assuming the enzyme activity at the optimum reaction temperature was 100%. Then, an Arrhenius plot [log V (logarithm of percent residual activity) vs. reciprocal of temperature in Kelvin (1000/T)] was used to calculate the activation energy (Ea) for xylanase, as illustrated in Eq. (5):

5 $$\mathrm{Ea}/\mathrm{R}=\mathrm{slope}$$

Next, samples were preincubated at temperatures ranging from 40 to 60 °C for up to 120 min to investigate the thermal stability of xylanase forms. Samples were first obtained at various times and tested for activity under conventional assaying settings. Then, the residual activity was determined, using the enzyme activity at 0 min of incubation as a starting point.

### Isolation and sequencing of the xylanase-encoding gene

#### Primer design and PCR amplification

First, total RNA was extracted using the RNeasy Mini Kit (Qiagen, cat. no. 74104c). Then, the ReverAid First Strand cDNA synthesis kit was used for cDNA synthesis (Thermo Scientific cat. no. K1621). All experiments were conducted according to the manufacturer’s instructions. Subsequently, the PCR reaction was performed using a GeneAmp PCR System 2400 thermal cycler (Perkin-Elmer Norwalk, Connecticut, USA). Primer sequences (forward primer: xynC-F 5′ ATGGTTCGTCTTACTGTTCTTGC3′ and xynC-R reverse primer: 5′TTATAAGGCGGAGATAATTGC3′) were first designed using cDNA from the *A. terreus* strain BCC129 xylanase family 10 (complete cds GenBank ID: DQ087436.1). The PCR protocol started with a 5 min denaturation at 94 °C, followed by 35 cycles of 30 s at 94 °C, 30 s at 48 °C, and 1 min at 72 °C. A final extension of 7 min at 72 °C was then performed. Finally, electrophoresis, visualization, and sequencing procedures were conducted using a similar internal transcribed spacer (ITS) region gene, except the xynC-F and xynC-R primers that were used in sequencing.

### Secondary structure prediction of xylanase from the A. terreus strain RGS.Eg-NRC

Primary xylanase protein sequences of the *A. terreus* strain RGS.Eg-NRC were obtained from the NCBI protein sequence database. Then, BLASTp was used to find the template structure with the closest homology to the xylanase model in the Protein Data Bank (PDB) database, using the xylanase sequence as an input query. Subsequently, the target and template sequences were multiply aligned using the clausal omega algorithm with default parameters, after which the secondary structure was predicted using the DSSP program [33].

### Molecular docking

#### Protein receptors preparation

The PyMOL software was used to process the 3D structure of xylanase by eliminating water molecules, ions, and existing ligands from the protein molecule. The insertion of hydrogen atoms to the receptor molecule was then carried out using AutoDock Vina software 7′s MG tools.

#### Preparation of ligands

The structure data format (SDF) of the substrate (xylan) was drawn by BIOVIA draw18.1 version, then, it was converted to a mol2 chemical format using an Open Babel. The non-polar hydrogen molecule was merged with the carbons, polar hydrogen charges of the Gasteiger-type were assigned, and the internal degrees of freedom and torsions were set to zero. The substrate molecule was converted to the dockable *pdbqt* format using the AutoDock tools.

### Docking studies

Docking studies were aimed at exploring the binding mode of the xylan as substrate and the 3-D model of xylanase protein using AutoDock tools 4.2. The macromolecule file was then saved in *pdbqt* format to be used for docking. Ligand centered maps were generated by the AutoGrid program with a spacing of 0.375A° and grid dimensions of 90A° × 90A° × 90A°. Grid box center was set to the coordinates (0.074, 0.083, −0.013) in (x, y, z) format, respectively. Polar H charges of the Gasteiger-type were assigned, and nonpolar-H atoms were merged with the carbons, and internal degrees of freedom and torsions were set.

### Visualization

Analysis of the 2-D hydrogen-bond interactions of the xylanase-substrates structure was performed by the Discovery Studio 4.5 program. It depicts hydrophobic bonds, hydrogen bonds, and their bond lengths in each docking pose graphically.

### Hydrolysis study (production of xylooligosaccharides)

The ability of the produced enzyme to hydrolyze beech wood xylan polymer was tested in reaction mixture consisted of 2 ml enzyme solution (5U/ml) and 2 ml 2% (w/v) xylan (in 50 mmol/l phosphate buffer pH 5.8) and carried out at 40 °C for 1, 2, 3, 4, 5, 6, 7, 8, 17, 20, 24, 26, and 28 h of incubation. The reactions were stopped by boiling for 10 min after 30 min. The amounts of reducing sugar of hydrolysis products were detected using DNSA methods. Hence, the hydrolysis product patterns were determined by thin-layer chromatography (solvent system and spraying agent as in [34].

### Investigating of antioxidant activity

Following a previously reported protocol [35], the assays of DPPH radical scavenging, reducing power ability, ABTS radical scavenging percentage using the antioxidant reference standards of BHT was determined for the investigated xylooligosaccharides, while the results of FRAP assay were presented in μM Trolox/100 g dry matter.

### *In vitro* antitumor activity using Ehrlich ascites carcinoma cells (EACC)

The antitumor activity was evaluated according to [36, 37] with some modifications. The tumor line EACC was brought from the National Council Institute and immediately diluted with physiological saline. The viability of EACC cells was determined before incubation using the bright-line hemocytometer slide. Several concentrations of XOS (1.00, 2.00, 3.00, and 4.00 mg/ml) were incubated with EACC cells at 37 °C for 2 h in CO2 incubator. After that, the tubes were centrifuged at 1000 rpm for 5 min. A total of 10 μl of cells suspension was added to 80 μl saline and 10 μl trypan blue and mixed. The viability was calculated. Viable cells appeared as unstained bodies, while nonviable cells stained blue. After incubation, the viability of tumor cells was calculated. Vincrestine was used as a standard drug.

## Results

### Molecular identification of xylanase producing enzyme

**Fig. 1:**
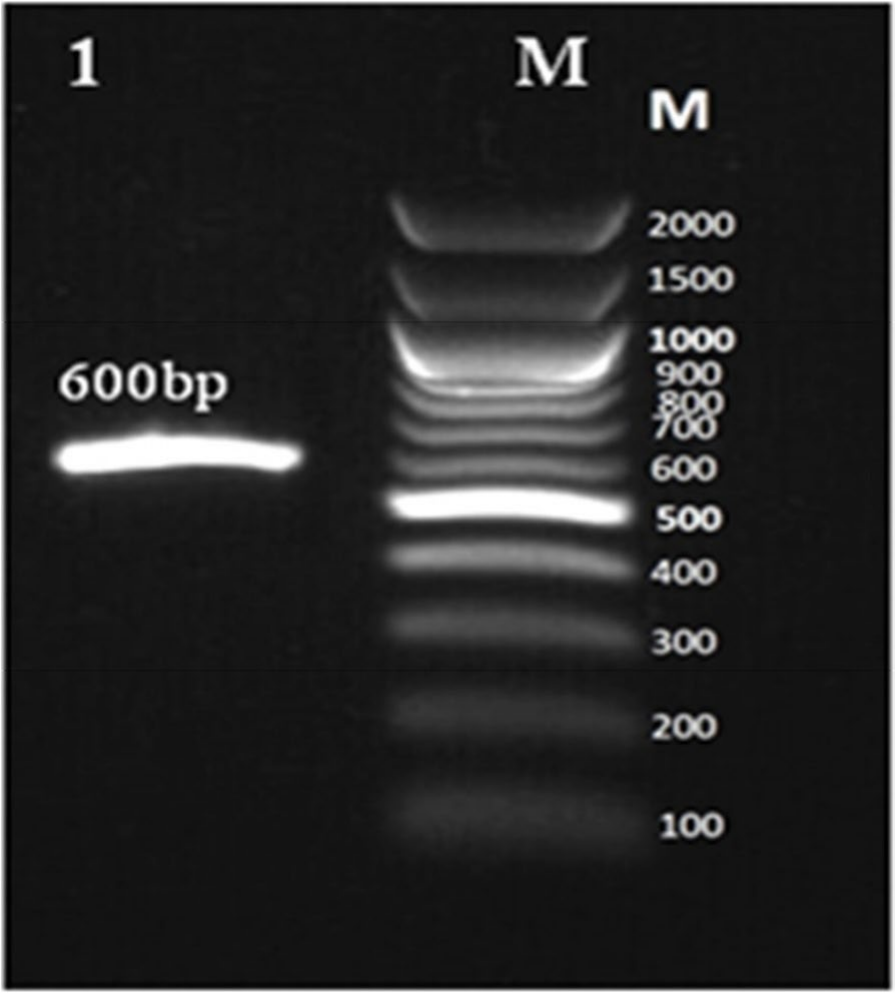
Agarose gel electrophoresis for PCR product (600 bp) of ITS region from *A. terreus* strain RGS. Eg-NRC; M, 100 bp DNA ladder (Invitrogen, California, USA)

**Fig. 2:**
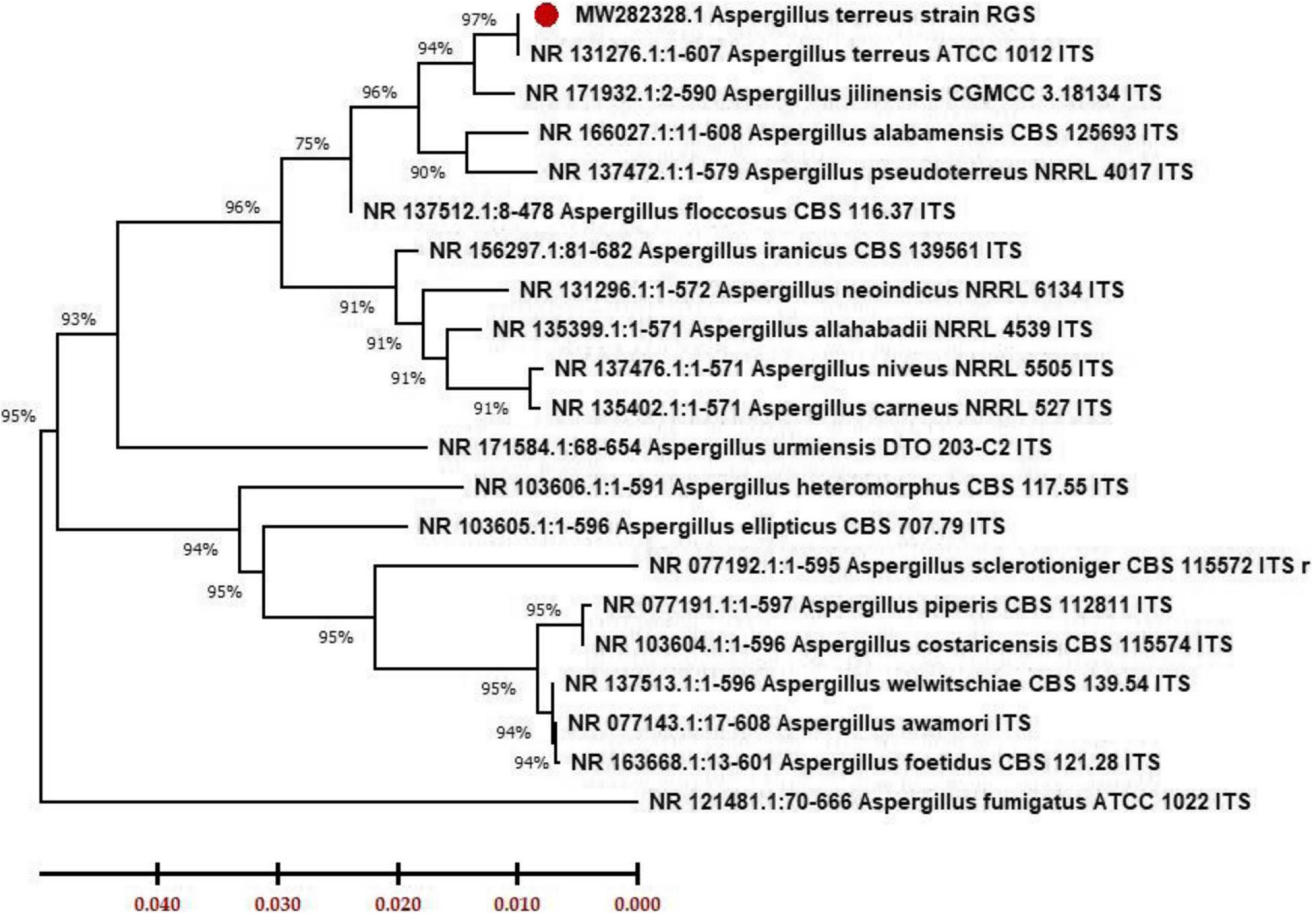
Phylogenetic tree of *A. terreus* strain RGS. Eg-NRC was inferred using the neighbor-joining method (MEGA X). The numbers on the nodes indicating the percent of bootstrap value

*Aspergillus* isolates were identified as *A. terreus* strain RGS.Eg-NRC after isolation and sequencing of the Internal transcribed spacer (ITS) region, which is 600 bp length (results are shown in Fig. 1). Then, it was deposited under accession no. MW282328. Partial nucleotide sequencing of the ITS region showed 97% identity with existing species in the GenBank database under the genus *Aspergillus*. However, from the blast results, the strain of RGS.Eg-NRC was closely related to only *A. terreus*. Subsequently, a phylogenetic tree was constructed using the isolates’ partial ITS region sequences. A phylogenetic tree includes representative strains of related species obtained using the neighbor-joining method. From the phylogenetic tree (Fig. 2), its result showed that RGS.Eg-NRC was closely related to *A. terreus* ATCC 1012ITS with 97% similarity.

### Cytotoxic activity tests

Cytotoxic activity tests (*in vitro* bioassay using human normal cell lines) were conducted to examine the safety of xylanase produced from *Ricinus communis* and establish its biological activities (antioxidant and antitumor). The samples were examined against the normal human epithelial cell line BJ1 (normal skin fibroblast), and the concentration range of the sample (unfermented *Ricinus communis*) ranged between 100 and 0.78 μg/ml. Results showed that while its LC_50_ was 50.9 μg/ml, its LC_90_ was 76.7μg/ml. Moreover, fermented *Ricinus communis* did not record any cytotoxic activity (Table 1), which means it is very safe.

**Table 1:** Cytotoxic activity of *Ricinus communis* (unfermented/fermented)

Sample code	LC_**50**_ (μg/ml)	LC_**90**_ (μg/ml)	Remarks
Unfermented *Ricinus communis*	50.9	76.7	100% at 100 ppm
Fermented *Ricinus communis* (xylanase)			1.2%
DMSO (dimethyl sulfoxide)			1% at 100 ppm
Negative control			0%

LC50, lethal concentration of the sample which causes the death of 50% of cells in 48 h. LC90, lethal concentration of the sample which causes the death of 90% of cells in 48 h

### Optimization of xylanase production

Subsequently, the production of *A. terreus* xylanase was optimized using two steps:

- One-variable-at-a-time experiment
- Statistical designs

#### One variable at a time

**Fig. 3:**
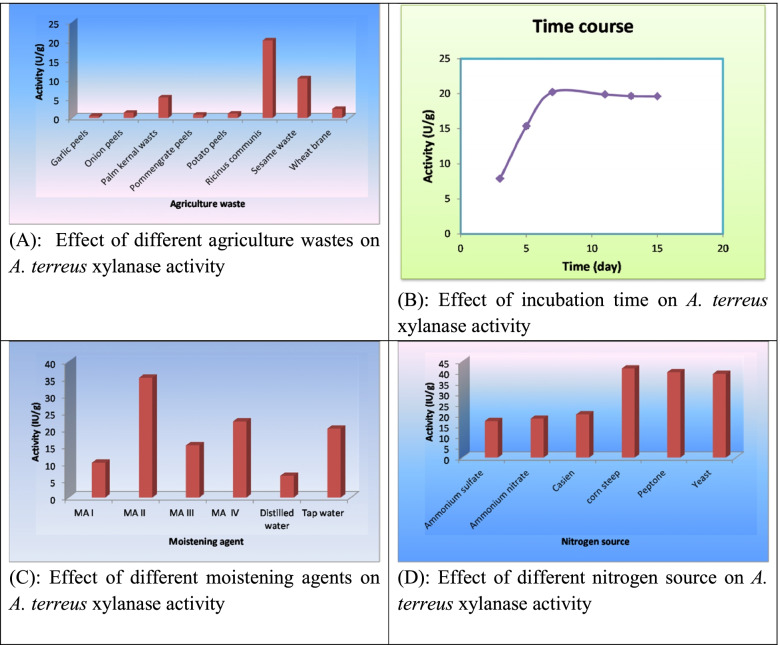
Effect of different factors on *A. terreus* xylanase activity Statistical optimization for the production of xylanase. **A** Effect of different agriculture wastes on *A. terreus* xylanase activity. **B** Effect of incubation time on *A. terreus* xylanase activity. **C** Effect of different moistening agents on *A. terreus* xylanase activity. **D** Effect of different nitrogen source on *A. terreus* xylanase activity

Different factors were used to study their effect on xylanase production from *A. terrus*. First, eight different agricultural wastes (garlic peels, onion peels, palm kernel wastes, pomegranate peels, potato peels, *Ricinus communis*, sesame waste, and wheat brane) were collected for xylanase production, after which *A. terrus* was grown using these agricultural wastes. Of all of these substrates, while *Ricinus communis* gave the highest xylanase yield (15.36 IU/g), garlic peels gave the lowest activity (0.5 U/g) (Fig. 3a). Subsequently, the time course of xylanase production by the SSF was studied. The results in Fig. 3b show that xylanase was maximally produced after 7 days of fermentation (yield of 20.23 U/g). Furthermore, results showed that substituting tap water with four different mineral salt solutions as moisturizing agents improved xylanase activity to 35.22 U/g d, and this result was obtained from MA II (Fig. 3c). Investigations also revealed that the best productivity was observed by replacing the constituent mixture of nitrogen sources with an equal value of each organic and inorganic nitrogen source constituent. Here, we observed that the productivity further increased to 41.66 U/g ds (Fig. 3d). Additionally, using varying corn concentration steps (0.2, 0.4, 0.8, 1, 1.4, and 1.6%), the productivity increased to 55.95 U/g ds (data not shown) as the concentration increased to reach (1.4%). However, after supplementing with 4.5% (w/v) of different sugar additives, significant effects on xylanase activity were further observed, with an even higher yield of 66.88 U/g ds of Glucose (data not shown).

### Statistical optimization of xylanase production

Many researchers believe that since a statistical optimization model for the fermentation process can overcome the limits of old empirical methods, it is more important for optimizing xylanase output. The statistical approaches adopted for xylanase optimization are as follows:

#### The Plackett–Burman design

PBD was used to investigate the relative interaction and the variables of different parameters for culture processing. Eleven trials for seven variables (Table 2) were used to first clarify the wide variation in the production of xylanase from 66.88 to 116.95 U/g. Results showed the great influence of the different factors in the fermentation process, with the highest value of the xylanase (116.95 U/g) being produced in trial 1, comprising the following: *Ricinus communis* waste (3 g/flask), corn steep (1%), KH_2_PO_4_ (6.5 g/l), glucose (4%), moistening agent (9 ml), inoculum (3 ml), and an incubation period of 9 days. Contrastively, the lowest value of xylanase (24.79 U/g) was produced in trial 6, comprising *Ricinus communis* waste (7 gm), corn steep (1%), KH_2_PO_4_ (8.5 gm/l), glucose (4%), moistening agent (7 ml), inoculum (3 ml), and an incubation period of fi5ve days.

**Table 2:** Plackett-Berman experiment coded levels and real values

Trial	*Ricinus communis*	Corn steep liquor	KH_2_PO_4_	Time	Inoculum size	Moistening agent	Glucose	Activity
1	3 (−)	1 (−)	6.5 (−)	9 (+)	3 (+)	9	4	116.95 ± 0.69
2	7 (+)	1 (−)	6.5 (−)	5 (−)	1 (−)	21	5	48.08 ± 0.85
3	3 (−)	1.8 (+)	6.5 (−)	5 (−)	3 (+)	3	5	59.48 ± 0.06
4	7 (+)	1.8 (+)	6.5 (−)	9 (+)	1 (−)	7	4	47.41 ± 0.08
5	3 (−)	1 (−)	8.5 (+)	9 (+)	1 (−)	3	5	55.33 ± 0.05
6	7 (+)	1 (−)	8.5 (+)	5 (−)	3 (+)	7	4	24.80 ± 0.25
7	3 (−)	1.8 (+)	8.5 (+)	5 (−)	1 (−)	9	4	86.78 ± 0.61
8	7 (+)	1.8 (+)	8.5 (+)	9 (+)	3 (+)	21	5	83.96 ± 0.36
9	5 (0)	1.4 (0)	7.5 (0)	7 (0)	2 (0)	10	4.5	66.88 ± 0.04
10	5 (0)	1.4 (0)	7.5 (0)	7 (0)	2 (0)	10	4.5	66.88 ± 0.04
11	5 (0)	1.4 (0)	7.5 (0)	7 (0)	2 (0)	10	4.5	66.88 ± 0.04

**Fig. 4:**
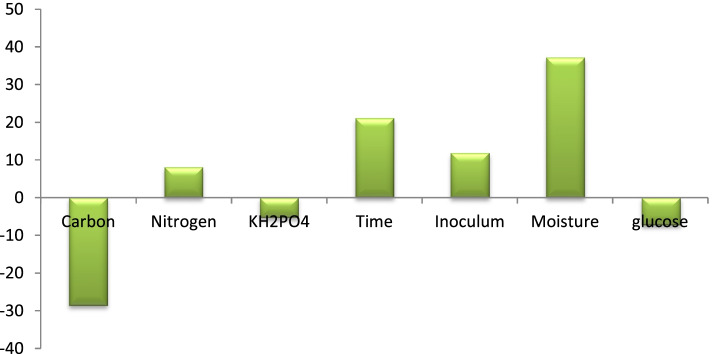
Main effects of independent variables on xylanase production according to the results of the PBD

The main effects of the investigated parameters on xylanase production are estimated and visually depicted in Fig. 4. Investigations revealed that while the examined factors, carbon, KH_2_PO_4_, and glucose, had negative effects, the nitrogen source, time, inoculum, and moistening agent (moisture) had positive effects. The confidence level, *P*-effect, and t-test of the statistical analysis of the PBD are indicated in Table 3. Therefore, since the *P*-value for the variables, carbon, time, and the moistening agent showed a high significant level, they were selected for further optimization.

**Table 3:** A statistical analysis of the Plackett-Burman design shows coefficient values, effect, *t*- and *P*-values for each variable on the xylanase study

	Coefficients	Standard error	*t*-Stat	*p*-value	Confidence level (%)
Intercept	119.5084119	6.889719517	17.3459038	0.000417549	
*Ricinus communis*	−14.58268826	0.296461368	−49.18916874	1.85019E^−05^	100
Corn steep	10.14870896	1.157487177	8.767880251	0.003124743	99.688
KH_2_PO_4_	−2.632637626	0.462994871	−5.686105379	0.010781161	98.922
Time	3.521502946	0.154331624	22.81776646	0.000184356	99.9816
Inoculum size	5.949054293	0.462994871	12.8490717	0.001017339	99.899
Moistening agent	3.718916414	0.092598974	40.16152929	3.39681E^−05^	100
Glucose	−22.14766313	0.997321474	−22.20714555	0.000199909	99.9801
The model’s summary					
Multiple R			0.99956197		
*R*^2^			0.99912412		
Adjusted *R*^2^			0.99708041		
Standard error			1.30954725		

The equation below shows a first-order model that describes the relationship between the seven components and xylanase activity:

$${Y}_{\mathrm{activity}}=119.5084119-14.58268826\ {\mathrm{X}}_1+10.14870896\ {\mathrm{X}}_2-2.63264\ {\mathrm{X}}_3+3.521503\ {\mathrm{X}}_4+5.949054\ {\mathrm{X}}_5+3.718916414\ {\mathrm{X}}_6-22.14766313\ {\mathrm{X}}_7$$

#### Central composite design

The most effective factors’ optimal amounts (carbon, moistening agent, and time) arising from PBD were further analyzed by applying RSM, involving CCD in 20 trials (Table 4). The culture medium contained *Ricinus communis* waste (3 g/flask), corn steep (1%), KH_2_PO_4_ (6.5 g/l), glucose (4%), moistening agent (9 ml), and inoculum 3 ml. Experiments were conducted for an incubation period of 9 days as the central point of the CCD3.

**Table 4:** Examined concentration of the key variables and results of CCD experiment

Trial no.	*X*_1_*Ricinus communis*	*X*_2_ Moistening agent	*X*_3_ Time	Activity (IU/g)	Predicted (IU/g)	Residual
1	(−)2	(−)4	(−)7	140 ± 1.036	136.6638	3.3362
2	(+)4	(−)8	(−)7	31 ± 0.69	37.86154	−6.86154
3	(−)2	(+)8	(−)7	139 ± 0.36	152.2008	−13.2008
4	(+)4	(+)16	(−)7	10 ± 0.58	3.150242	6.849758
5	(−)2	(−)4	(+)15	174.05 ± 0.23	195.4369	−21.3863
6	(+)4	(−)8	(+)15	120 ± 0.89	118.6143	1.3857
7	(−)2	(+)8	(+)15	211 ± 0.95	204.9636	6.0364
8	(+)4	(+)16	(+)15	65 ± 1.06	71.88248	−6.88248
9	(−2) 1	(0) 3	(0) 11	245 ± 0.25	236.2326	8.7674
10	(+2) 5	(0) 15	(0) 11	5 ± 0.68	6.088892	−1.08889
11	(0) 3	(−2) 3	(0) 11	128 ± 1.22	121.7481	6.2519
12	(0) 3	(+2) 15	(0) 11	109 ± 0.88	110.0048	−1.0048
13	(0) 3	(0) 9	(−2) 3	45 ± 1.30	43.90515	1.09485
14	(0) 3	(0) 9	(+2) 19	181 ± 1.66	174.4156	6.5844
15	(0) 3	(0) 9	(0) 11	116 ± 0.28	113.9783	2.0217
16	(0) 3	(0) 9	(0) 11	116 ± 0.28	113.9783	2.0217
17	(0) 3	(0) 9	(0) 11	116 ± 0.28	113.9783	2.0217
18	(0) 3	(0) 9	(0) 11	116 ± 0.28	113.9783	2.0217
19	(0) 3	(0) 9	(0) 11	116 ± 0.28	113.9783	2.0217
20	(0) 3	(0) 9	(0) 11	116 ± 0.28	113.9783	2.0217

R-square, 0.98622; adjusted R-square, 0.972439. *P*-value: 2.69E-07

Table 4 lists the independent variables, their coded matrices, and replies, including the experimental and projected values for xylanase activity. We observed a variation in enzyme yield over time, ranging from 5 to 245 IU/g during the 20 runs of the experiments. Notably, the highest level of the produced xylanase was 245 IU/g obtained in run 9, which indicated that the optimal levels of the tested variable were as follows: *Ricinus communis* waste (1 g/flask), moistening agent (3 ml), and incubation period (11 days).

Furthermore, the model’s accuracy’s coefficient (R2) was 0.9862, indicating that the independent variables accounted for 98.62% of the response variability. As a result, the current R2 value confirmed the validity of the existing model for xylanase production, including a good connection between the experimental and theoretical values (Table 4).

The equation below was used to analyze the association between variables and response using a second-order polynomial equation:

$${Y}_{\mathrm{Activity}}=243.0467-121.621\ {\mathrm{X}}_1+13.42214\ {\mathrm{X}}_2+6.255342\ {\mathrm{X}}_3+14.60489\ {{\mathrm{X}}_1}^2+0.052727\ {{\mathrm{X}}_2}^2-0.07528\ {{\mathrm{X}}_3}^2-4.42794\ {\mathrm{X}}_1{\mathrm{X}}_2+1.749367\ {\mathrm{X}}_1{\mathrm{X}}_3-0.18782\ {\mathrm{X}}_2{\mathrm{X}}_3$$

where *Y* represents the response and xylanase yields were represented using *X*_1_, *X*_2_, and *X*_3_.

**Fig. 5:**
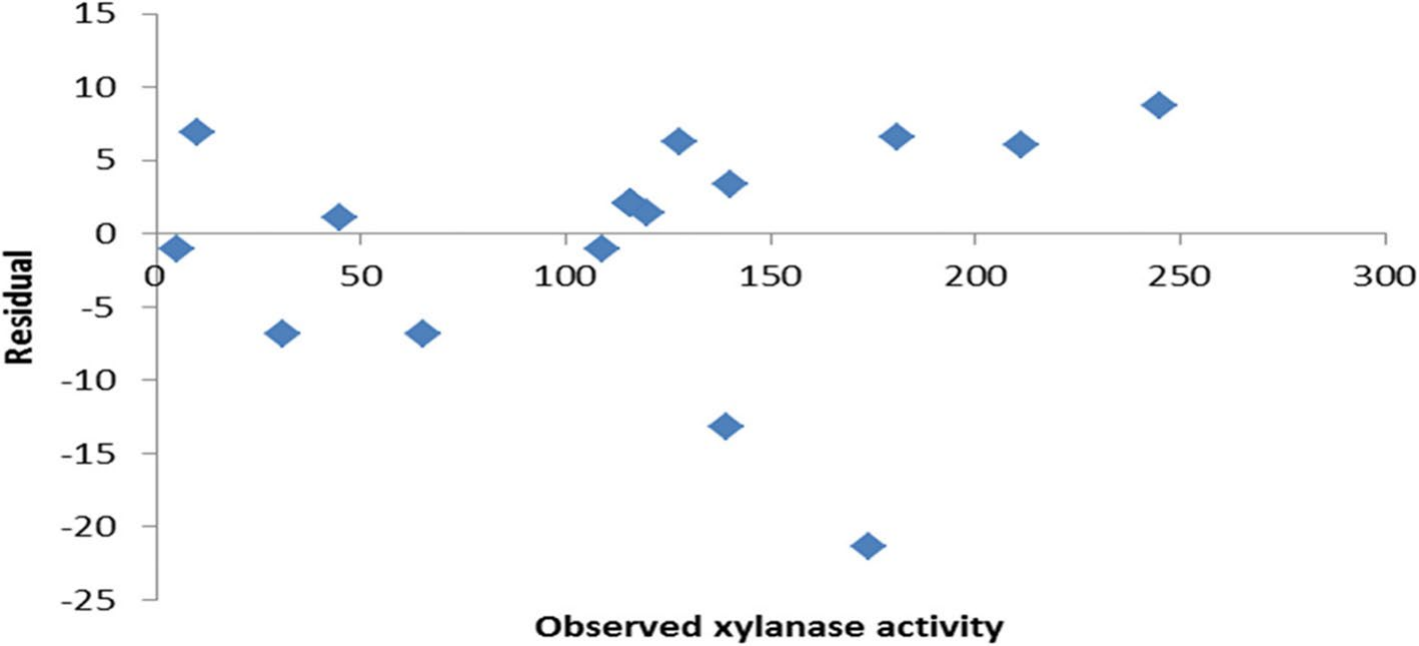
Residual plot of the observed-predicted values (residuals) versus the response (optimization process) of *A. terreus* xylanase

Subsequently, the *P-*value was employed to determine each coefficient’s magnitude, which revealed the pattern of the factors’ interactions. The statistical analysis of data (Table 5) indicated a high significant effect on xylanase production and smaller *P*-values (*P* < 0.05). Furthermore, the residual analysis (Fig. 5), which involved plotting the observed-predicted values (residuals) vs. the response (optimization process), revealed that the residuals formed a symmetrical pattern and were evenly distributed across the range, indicating that the average model was correct for all observed results.

**Table 5:** Analysis of CCD for xylanase activity

	*Coefficients*	*Standard error*	*t-Stat*	*p-value*
Intercept	243.0467	41.5702	5.846657	0.000245
X_1_	−121.621	23.62163	−5.14873	0.000604
X_2_	13.42214	5.499399	2.440655	0.037324
X_3_	6.255342	4.139276	1.511216	0.16502
X_1_^2^	14.60489	5.164777	2.827786	0.019794
X_2_^2^	0.052727	0.230607	0.228645	0.824256
X_3_^2^	−0.07528	0.135151	−0.55697	0.591127
X_1_X_2_	−4.42794	1.907266	−2.32162	0.045365
X_1_X_3_	1.749367	1.287861	1.358351	0.207422
X_2_X_3_	−0.18782	0.295456	−0.63568	0.540807

### Validation of the model

The proposed model’s validity was evaluated by predicting *A. terreus* xylanase production for each trial of the matrix. The experimental results in Table 4 show that the maximally observed xylanase production (245 U/gds) was very close to the predicted value (236 U/gds). Investigations also revealed that the optimum xylanase production reached 245 U/gds (12.1-fold) by applying OVAT, followed by statistical optimization for the SSF of *Ricinus communis* waste.

### Biochemical characterization of A. terreus strain RGS.Eg-NRC xylanase

#### Optimal pH and stability

**Fig. 6:**
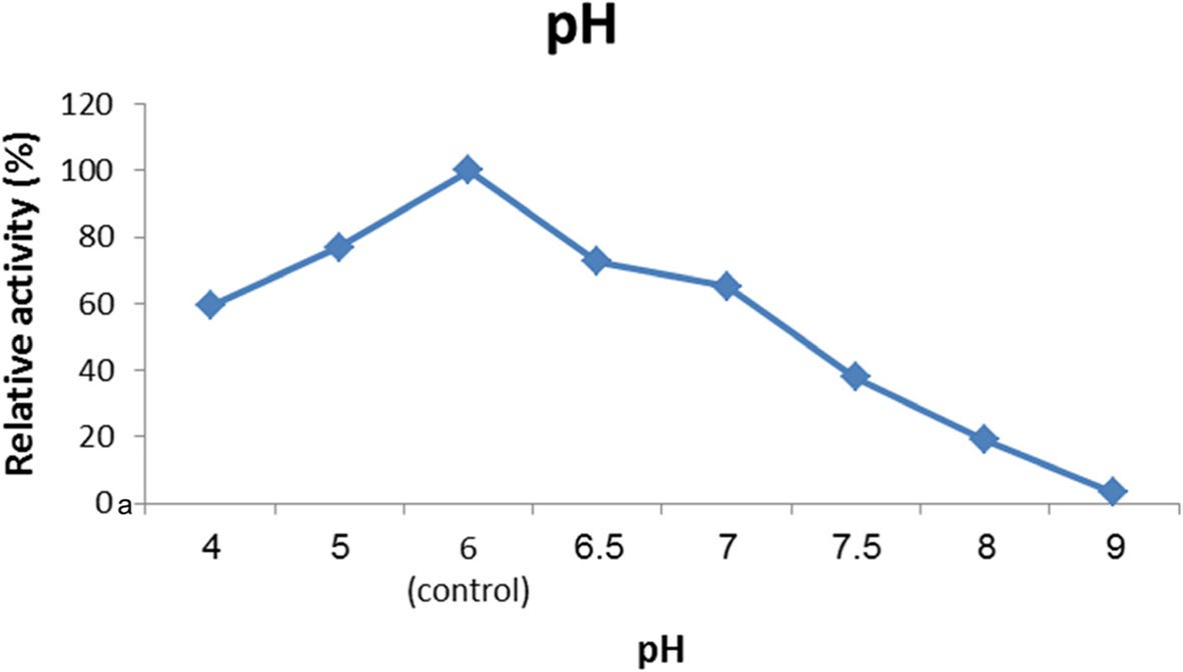
The effect of the reaction mixture’s pH on *A. terreus* strain RGS activity. Xylanase Eg-NRC. With 1% (w/v) xylan, the reaction was carried out at 50 °C

**Fig. 7:**
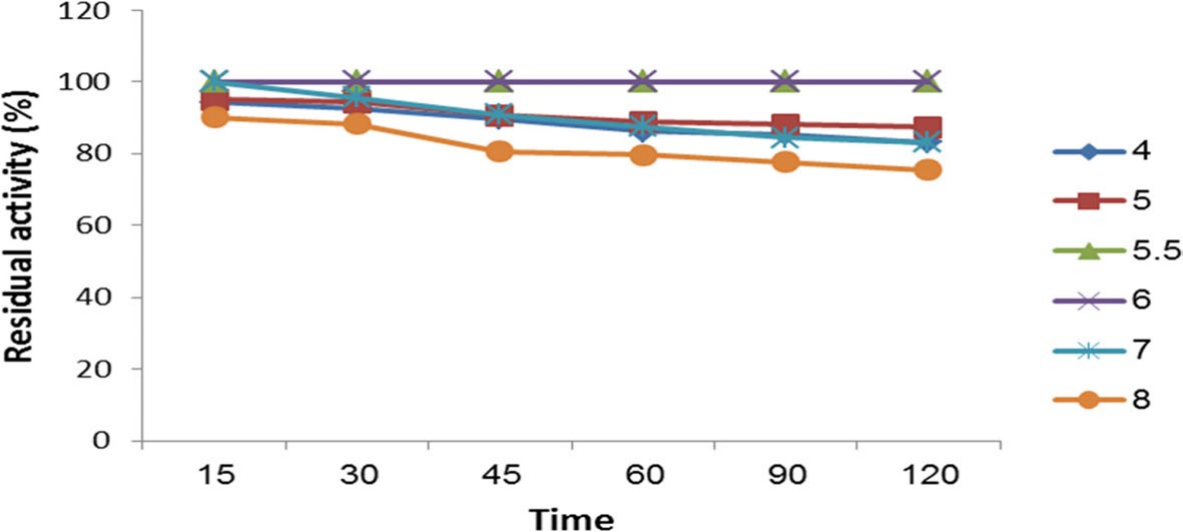
pH stability of *A. terreus* strain RGS. Eg-NRC xylanase. The enzyme solution was incubated at various pHs for various lengths of time, and the residual activity was evaluated under ideal circumstances

According to the data in Fig. 6, the optimum pH of the *A. terreus* xylanase was six. Figure 7 shows the pH stability of the enzyme under assay. Investigations revealed that while the enzyme was almost stable at pH 6 for 2 h, it was extremely stable at a pH range of 5 to 6. Following a 2-h incubation at this pH range, the enzyme lost 12.77% of its activity.

### Influence of temperature on the activity of enzymes

**Fig. 8:**
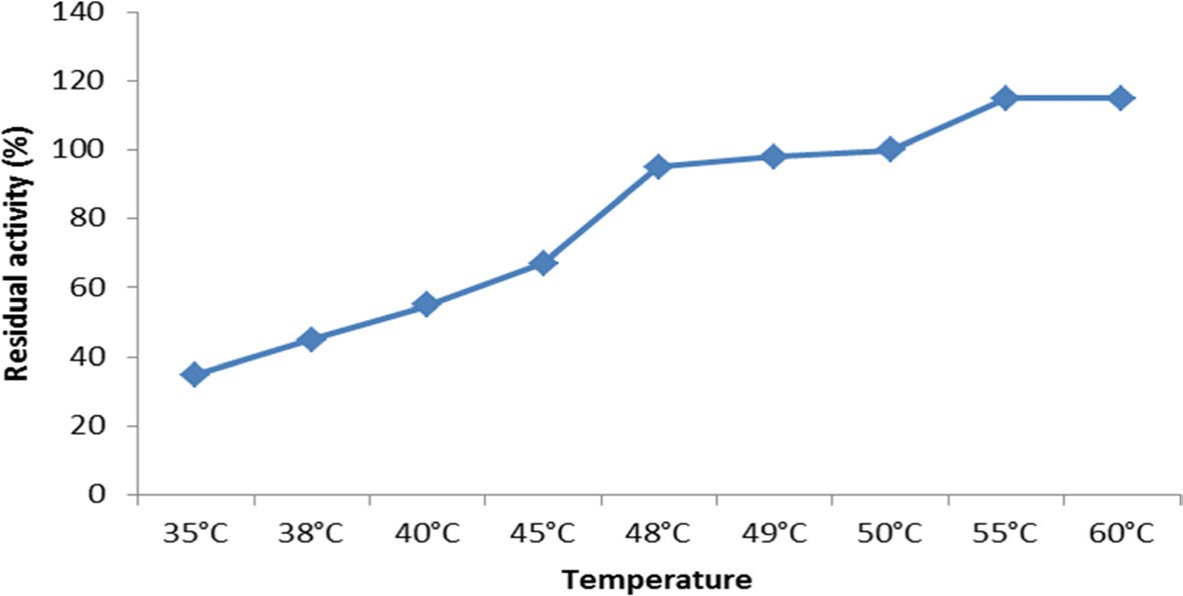
Effect of temperature of the reaction on the activity of *A. terreus* strain RGS. Eg-NRC xylanase. At varied temperatures, reactions with 1% (w/v) xylan were carried out at pH 6, 50 °C

The temperature dependency of *A. terreus* xylanase activity at temperatures ranging from 40 to 60 °C and at pH 6 is shown in Fig. 8. We observed that the activity of xylanase was stable at 60 °C, indicating that its optimum temperature was 55 °C. Therefore, a high temperature is recommended from a biotechnological standpoint because it improves conversion rates, reduces microbial contamination, and allows for increased substrate solubility.

#### Thermal stability

The Arrhenius equation is useful for calculating the activation energy and the efficiency of chemical reactions. When a reaction obeys the Arrhenius’ equation, a linear relationship is shown after graphing the log of residual activity against time, displaying a first-order kinetic reaction of the enzyme, whose gradient and intercept can then be used to calculate the activation energy (Ea). Xylanase formed a similar graph, which was consistent with this equation.

**Fig. 9:**
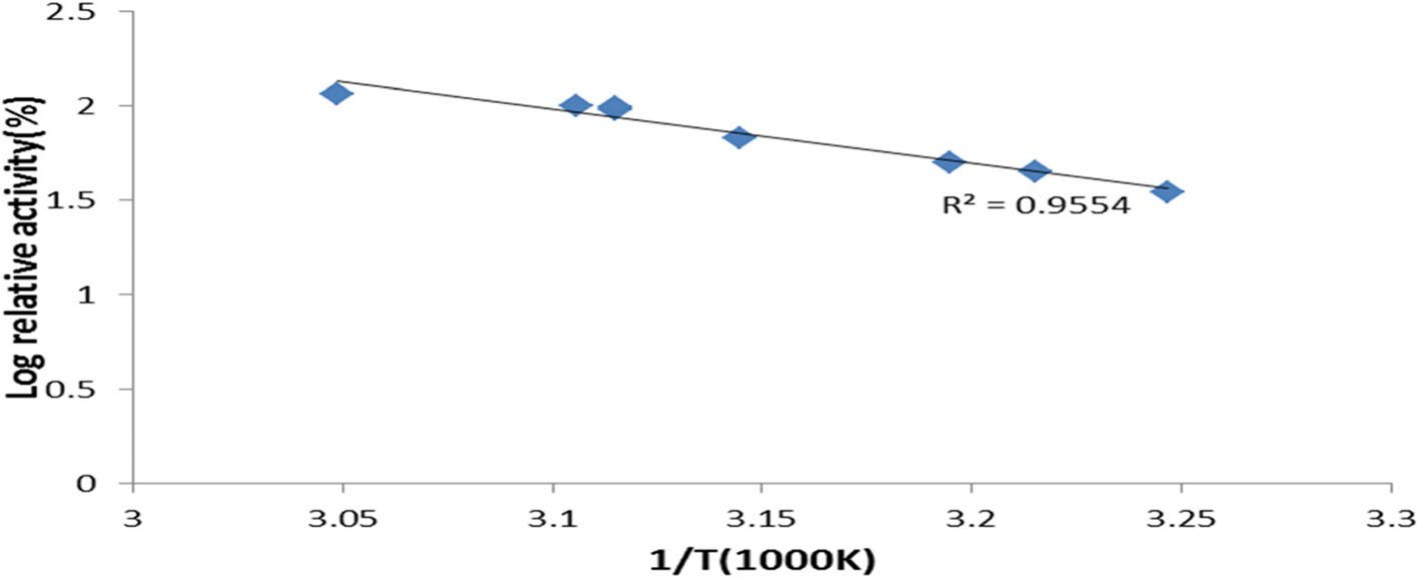
Arrhenius plots to calculate activation energy (Ea) for *A. terreus* xylanase

Subsequently, Arrhenius plots were used to calculate the catalysis’s activation energy (Ea) for the *A. terreus* xylanase (Fig. 9). For the Arrhenius plots of xylanase, the regression equation was *y* = −2.8772 + 10.901.

The results showed that the *E*_a_ of xylanase from *A. terreus* was 23.919 kJmol. The lower the value of Ea, the less energy it takes to conform to the enzyme-substrate complex’s active site. Therefore, because of these characteristics, and since it requires a low activation energy value, which will impact the total cost of industrial processing, *A. terreus* xylanase was considered more suitable for industrial applications.

**Fig. 10:**
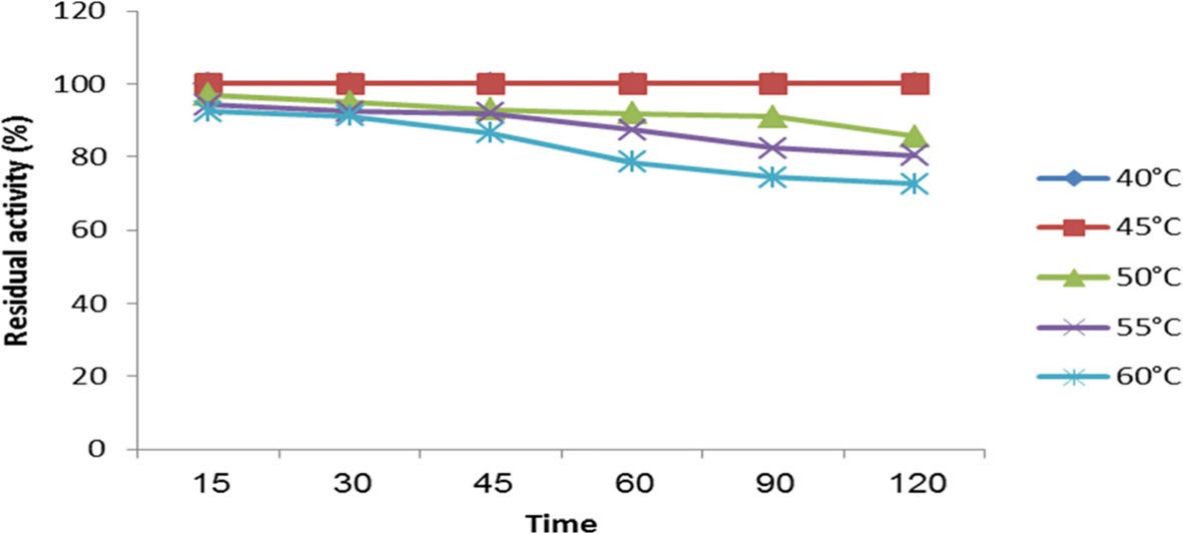
Temperature-stability profile for *A. terreus* xylanase

Additionally, Fig. 10 reveals that while the enzyme did not lose any activity after 120 min of incubation at 40 °C and 45 °C, 85.6% activity was preserved after 120 min of incubation at 50 °C. However, it lost roughly 13.5% of its activity after 120 min incubation at 60 °C.

### Hydrolysis property (production of xylooligosaccharides)

**Fig. 11:**
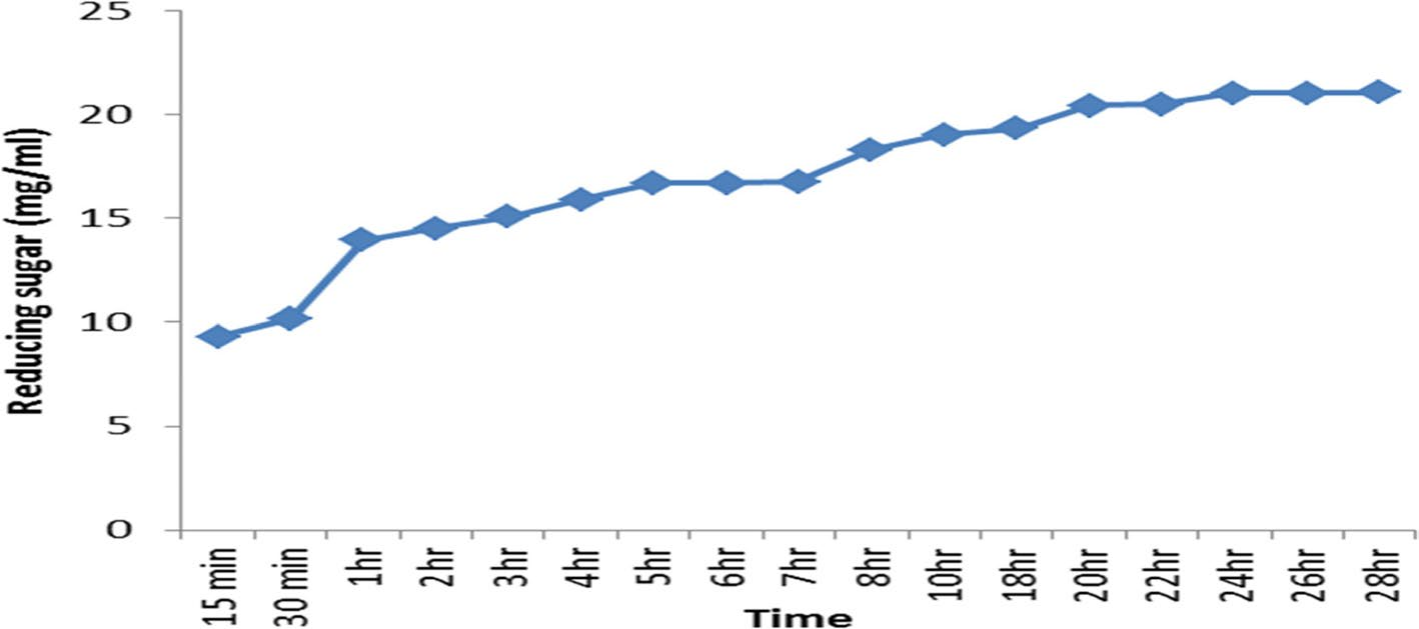
The released sugar produced from hydrolysis of xylan by *A. terreus* RGS xylanase at different times

**Fig. 12:**
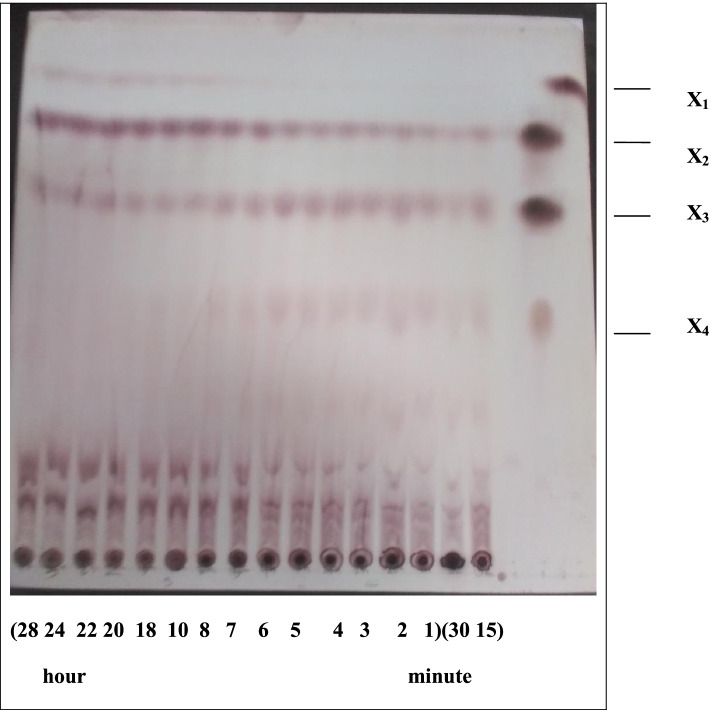
TLC plate of hydrolysis product of xylan by *A. terreus* xylanase at various periods. X1, mono; X2, Di; X3, Tri; X4, Tetra; lan 1, 15 min; lan 2, 30 min; and lan 3 to lan 14 are from 1 to 28 h

**Fig. 13:**
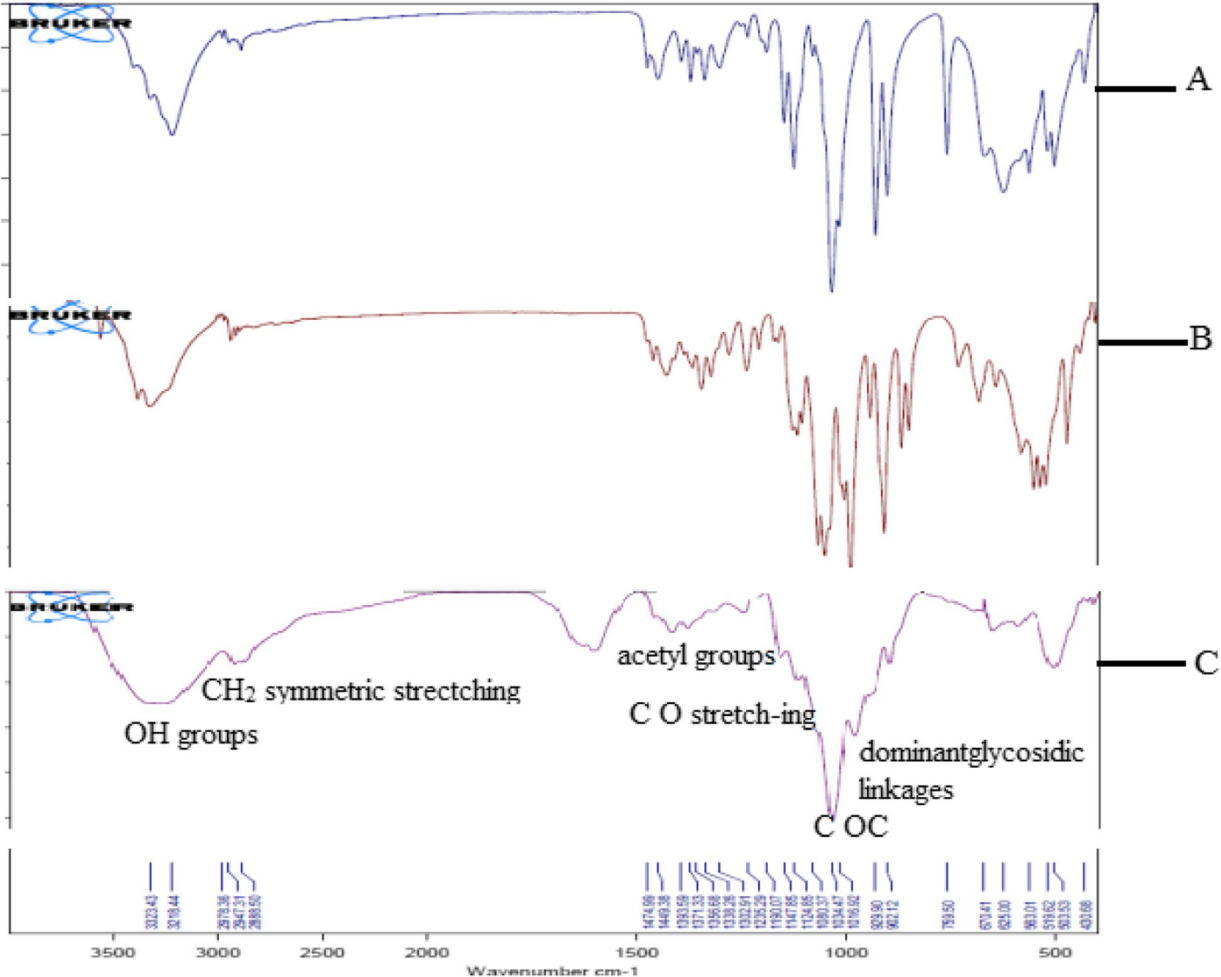
The spectrums of FTIR of the freeze-dried oligosaccharides products

Subsequently, the hydrolysis period of beech wood xylan by *A. terreus* xylanase was studied. The amount of reducing sugars released is shown in Fig. 11. Results showed that while the highest reducing sugar (21.08 mg/ml) was achieved after 28 h, other oligosaccharides of various sizes were detected by thin-layer chromatography (Fig. 12). Accordingly, FTIR was obtained from the hydrolysis of xylan. The applicability of the FTIR spectroscopy spectra to identify different functional groups in oligosaccharide samples has been proven. Hence, Fig. 13 shows the FTIR spectra of freeze-dried XOS products. The results showed that while the wideband near 3274 cm^1^ corresponded to the hydrogen-bonded OH group than water, all main carbohydrate components, the band detected at 2921 cm^1^ was attributed to the stretching vibrations of C–H bonds (CH_2_ symmetric stretching) [38]. Results also showed that the signals in 1417, 1382, 1246, and 1212 cm^−1^ were related to acetyl groups. Therefore, these bands were allocated to the single-bonded oxygen (C–O) stretching and symmetric CH_3_ bending vibrations. Notably, the region between 1150 and 920 was where xylan’s fingerprint was located. The C–O and C–C stretching and/or CO–H bending, including the glycosidic linkage (C–OC) contributions, was attributed to the region between 1150 and 920 cm^1^ in the xylan fingerprint. However, the greatest band at 1034 cm^1^ was assigned to the C–O and C–C stretching and/or CO–H bending and glycosidic linkage (C–OC) contributions [39], suggesting the preponderance of xylan oligosaccharides. Finally, the dominant glycosidic connections between xylose units in hemicelluloses were linked to the band at 897 cm^1^.

### Biological activity of xylooligosaccharides

#### Antioxidant capacity

Additionally, our study evaluated the antioxidant capacity of different XOS concentrations (0.5, 0.75, 1.00, 1.25, 1.50, and 2.00 mg/ml) using different assays (DPPH, reducing power ability, ABTS, and FRAP). BHT was used as a standard antioxidant agent. Results illustrated in Table 6 show that all investigated assays were similarly dose dependent, increasing gradually with increasing concentration. DPPH radical scavenging activity (%) ranged from 17.37 ± 0.14 to 82.79 ± 014% at concentrations 0.5 and 2.00 mg/ml, respectively. However, reducing power ability was evaluated by reading the absorbance at 700 nm, and results varied from 0.423 ± 0.013 to 1.024 ± 0.012 for the lowest (0.5 mg/ml) and the highest (2.00 mg/ml) concentrations. After the ferric reducing power capability investigations, results showed that the highest concentration of XOS (2.00 mg/ml) exhibited 1453 ± 19.67 μm Trolox/100g and gradually decreased to 346 ± 15.52 μm Trolox/100g using the lowest concentration of XOS (0.5 mg/ml). Moreover, the ABTS radical scavenging activity followed the same trend and increased gradually from 29.77 ± 0.29 to 80.57 ± 0.19% by increasing XOS concentrations from 0.5 to 2.00 mg/ml.

**Table 6:** Antioxidant capacity of different concentrations of xylooligosaccharide using different antioxidants assays

Concentrations	DPPH%	Reducing power (abso. at 700 nm)	FRAP (μm Trolox/100 g)	ABTS%
0.5 mg	17.37 ± 0.14	0.423 ± 0.013	346 ± 15.52	29.77 ± 0.29
0.75 mg	30.43 ± 0.12	0.535 ± 0.007	543 ± 12.66	42.64 ± 0.30
1.00 mg	43.92 ± 0.11	0.681 ± 0.020	774 ± 13.05	51.39 ± 0.24
1.25 mg	51.19 ± 0.21	0.844 ± 0.020	910 ± 16.62	63.18 ± 0.28
1.50 mg	72.33 ± 0.18	0.955 ± 0.019	1245 ± 17.50	72.38 ± 0.22
2.00 mg	82.79 ± 014	1.024 ± 0.012	1453 ± 19.67	80.57 ± 0.19
BHT	92.02 ± 0.74	1.69 ± 0.060	-	93.43 ± 0.33

All the values are the average of three independent experiments ± SD

#### In vitro antitumor activity

**Fig. 14:**
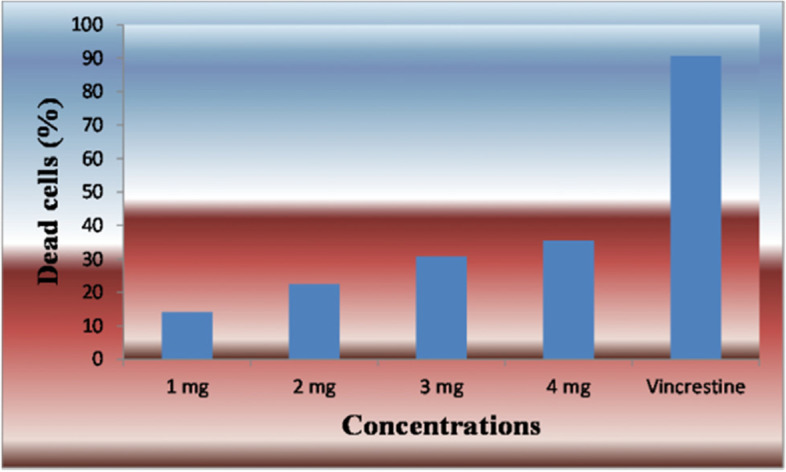
Effect of different concentrations of xylooligosaccharides (1.00, 2.00, 3.00, and 4.00 mg/ml) on the viability of EACC compared with vincristine

Figure 14 indicates that although different concentrations of XOS (1.00, 2.00, 3.00, and 4.00 mg/ml) exhibited a weak to moderate effect on the viability of Ehrlich ascites carcinoma cells, the activity of the cells was increased gradually by increasing the concentration of XOS. We also observed that while the dead cells (%) varied from 14.1 ± 0.79 to 35.61 ± 0.35 at 1 and 4 mg/ml, respectively, the standard antitumor drug (vincristine) recorded 90.65 ± 0.33% dead cells. Additionally, increasing the concentration of XOS to more than 4 mg/ml did not increase the dead cells of Ehrlich ascites carcinoma cells anymore. Based on these results, we surmise that the antitumor potential of XOS may likely be attributable to the efficient release of phenolic compounds, including its scavenging activity, which could alleviate the harmful effects of reactive oxygen species.

### Xylanase partial purification and sodium dodecyl sulfate-polyacrylamide gel electrophoresis

**Fig. 15:**
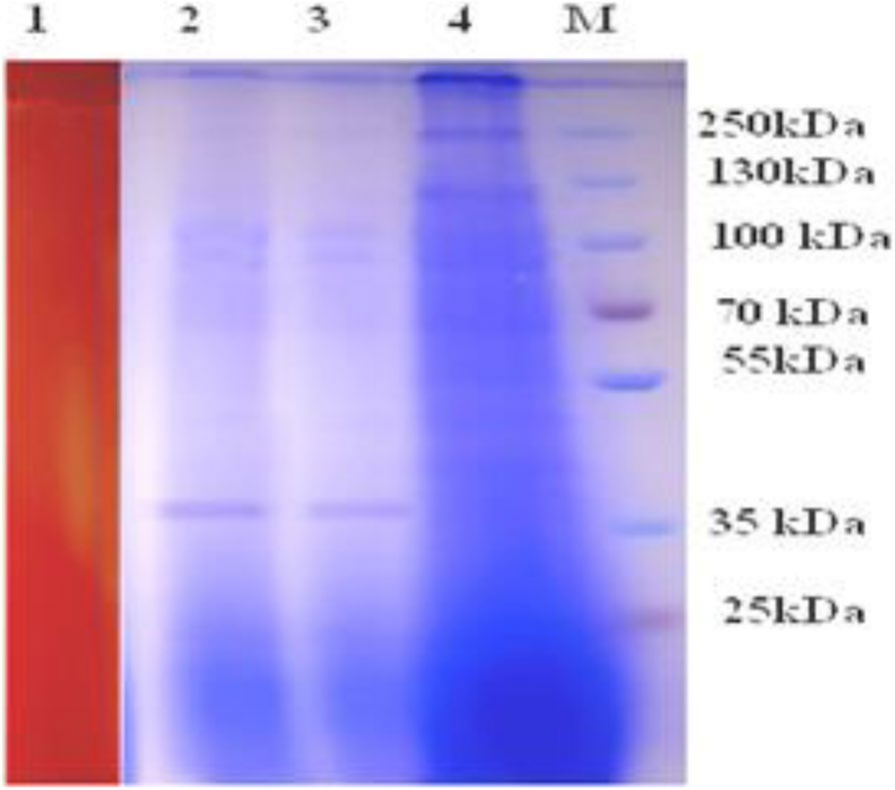
Zymogram of xylanase, 2-4 SDS-PAGE of partially purified xylanase from *A. terreus* RGS.Eg-NRC

Ethanol, acetone, and ammonium sulfate fractional precipitation achieved the crude enzyme partial preparation. A 60–70% ammonium sulfate fraction recovered 60.88%-specific activity with 3.9 U/mg protein. Thus, enzyme purification increased the activity by 31 folds. For zymogram analysis, the native PAGE was used. A clear zone on the agar gel revealed the presence of xylanase and helped localize the xylanase when the native PAGE gel was overlayered on the agar gel seeded with its substrate (xylan) (Fig. 15). During the enzyme preparation, zymogram analysis also indicated the existence of a single xylanase. Subsequently, the enzyme’s molecular weight was determined using SDS-PAGE. Partial purified enzymes had just one band, corresponding to a molecular weight of ~36 kDa (Fig. 15).

### Isolation and molecular characterization of the xynC gene from A. terreus RGS.Eg-NRC

**Fig. 16:**
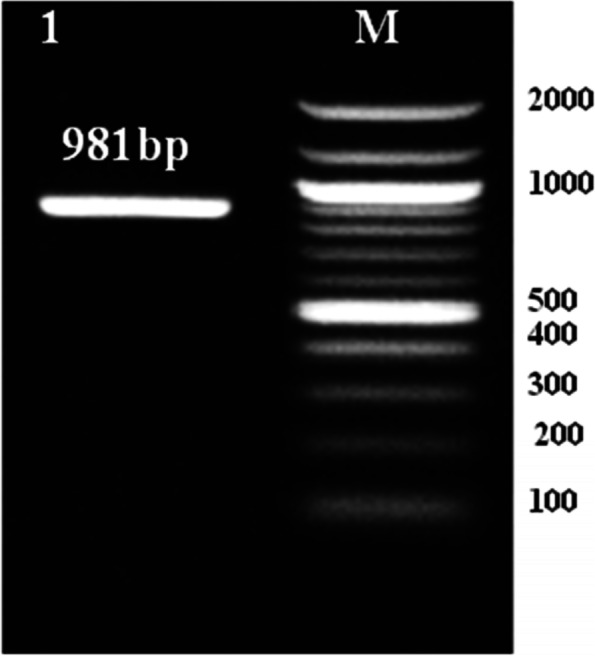
Agarose gel electrophoresis for PCR product of *xyn C* gene (in lane 1). Lane M, 100 bp DNA ladder (Invitrogen, California, USA)

The designed primers for the *xynC* gene were successfully amplified and confirmed to be 981 bp, as expected for the molecular weight of the total length of mRNA encoding the xylanase, using cDNA as a template (Fig. 16). The assembled sequence was subsequently subjected to a BLAST search on NCBI against the available sequences deposited in the NCBI database. BLAST analysis revealed an open reading frame (ORF) comprising 981 bp in the full-length xylanase gene (*xynC*) that encodes a protein consisting of 326 amino acids. Therefore, the gene sequence for the *xynC* gene was submitted to GenBank (accession number: LC595779.1).

**Fig. 17:**
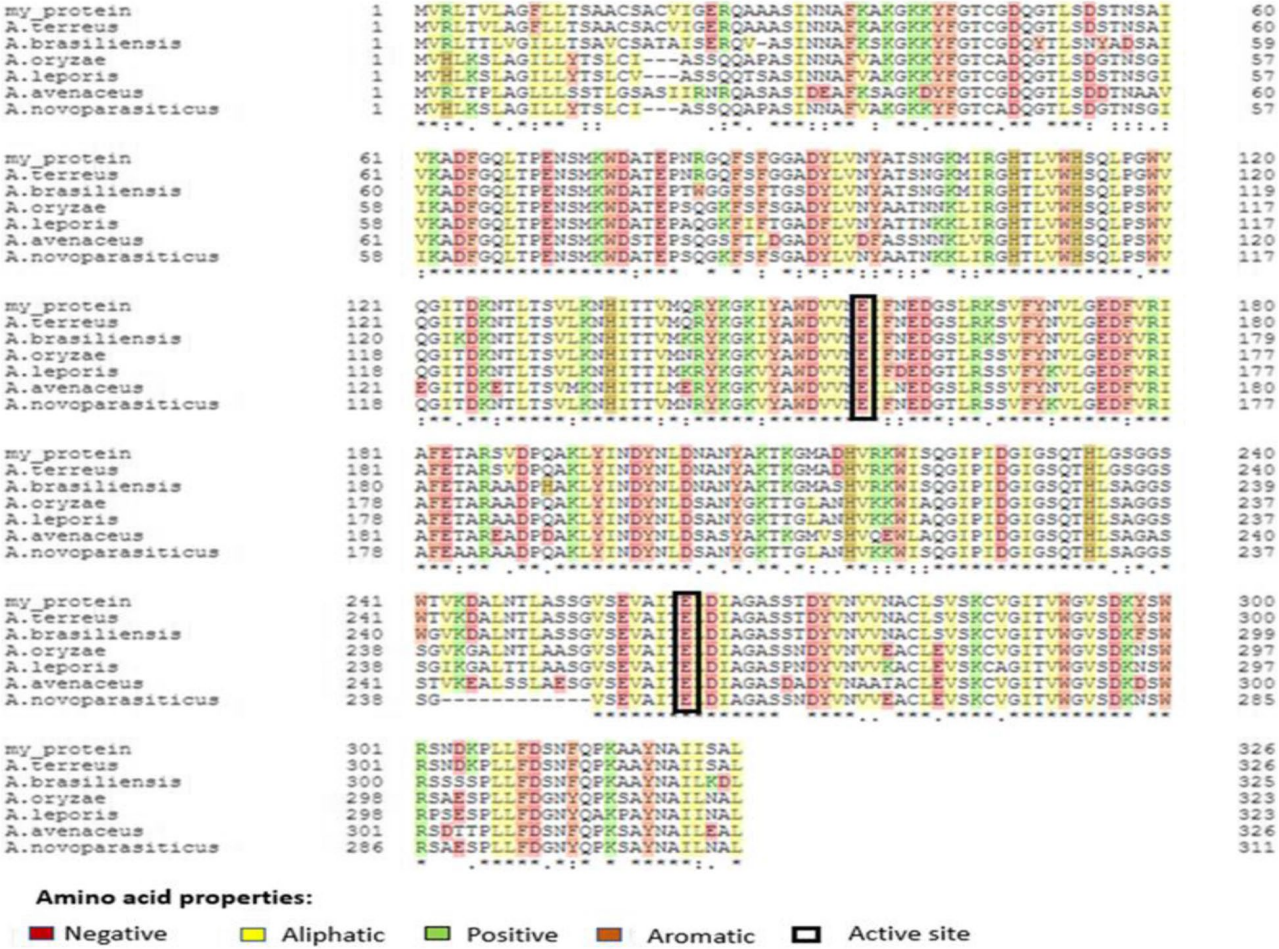
Multiple sequence alignment of amino acids for xylanase gene from *A. terreus* strain RGS. Eg-NRC (xyl) gene and other xylanase genes

**Fig. 18:**
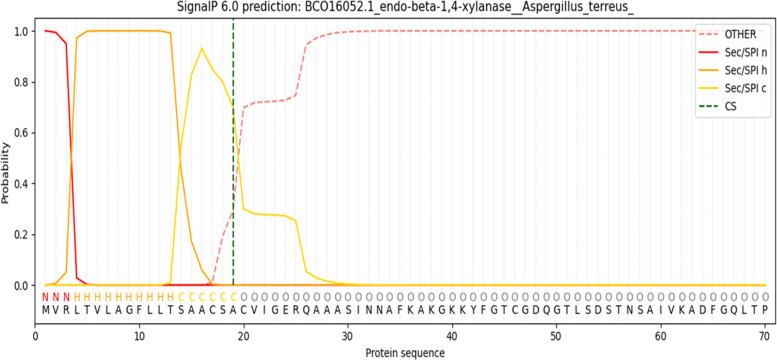
Signal IP prediction of amino acids for xylanase gene from *A. terreus* strain RGS. Eg-NRC (xyl) gene and other xylanase genes

**Fig. 19:**
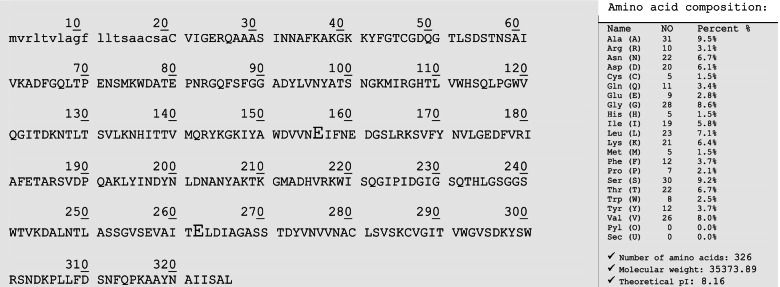
Complete amino acid sequence of xylanase gene from *A. terreus* strain RGS. Eg-NRC with amino acid composition

Next, the nucleotide sequence of *xlnC* was translated to its deduced amino acids (understudied protein) and then aligned against six other retrieved xylanases from the reviewed protein database, UniProt (Fig. 17). Xylanase C (understudied protein) had a 100% similarity with the *A. terreus* QOCMB8 strain. Furthermore, amino acid residues ranging from 11 to 326 were reported by the InterProScan server (EMBL) to belong to the glycosyl hydrolase family 10 (GH10). Subsequently, signal peptides and their cleavage sites were checked by the SignalP-6.0 server, and the results included residues ranging from 1 to 19 (Fig. 18). Therefore, we searched for O-glycosylation and N-glycosylation sites using the NetNGlyc 4.0 and GlycoEP server, but did not find any potential O-glycosylation and N-glycosylation sites. Amino acid composition and physicochemical properties are illustrated in Fig. 19. Investigations revealed the active site of XynC at two positions (Glu 156 and Glu 262), where E156 was the general acid/base residue and E262 was the catalytic nucleophile. Finally, while the theoretical molecular weight of a xyl protein was calculated as 35.3 kDa, the isoelectric pH was 8.16 (Fig. 19).

**Fig. 20:**
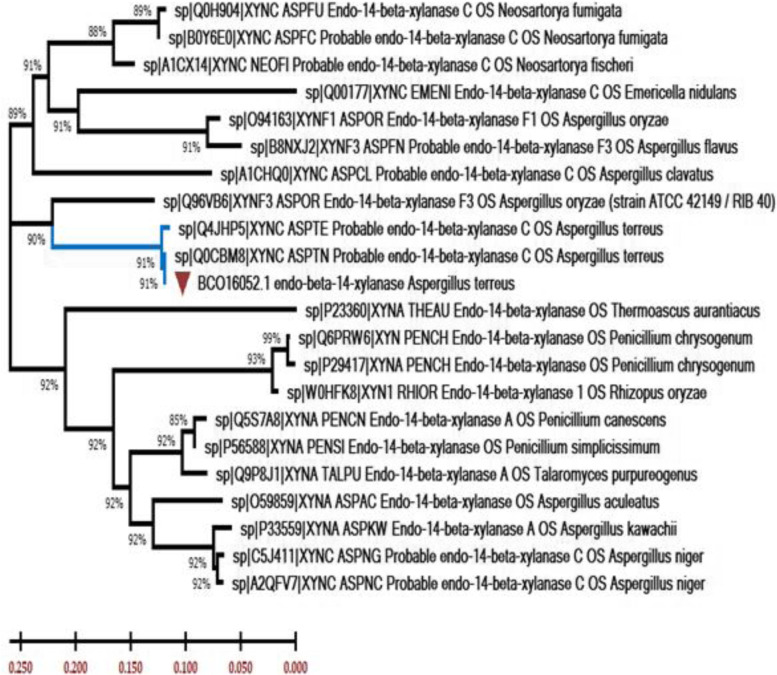
Phylogenetic tree of xylanase proteins from *A. terreus* strain RGS. Eg-NRC was inferred using the neighbor-joining method (MEGA X)

Subsequently, phylogenetic analysis was conducted to place *xyl* among the known xylanase family members (Fig. 20). All 21 curated xylanase proteins retrieved from the reviewed UniProt protein database were related to various organisms and were selected from the database for the phylogenetic analysis. The dataset included xylanase proteins from fungi. Final comparative analyses indicated that xylanase exhibited 91% sequence similarity to homologous fungal xylanase proteins (e.g., *Aspergillus* sp.)

### Secondary structure prediction of xylanase

**Fig. 21:**
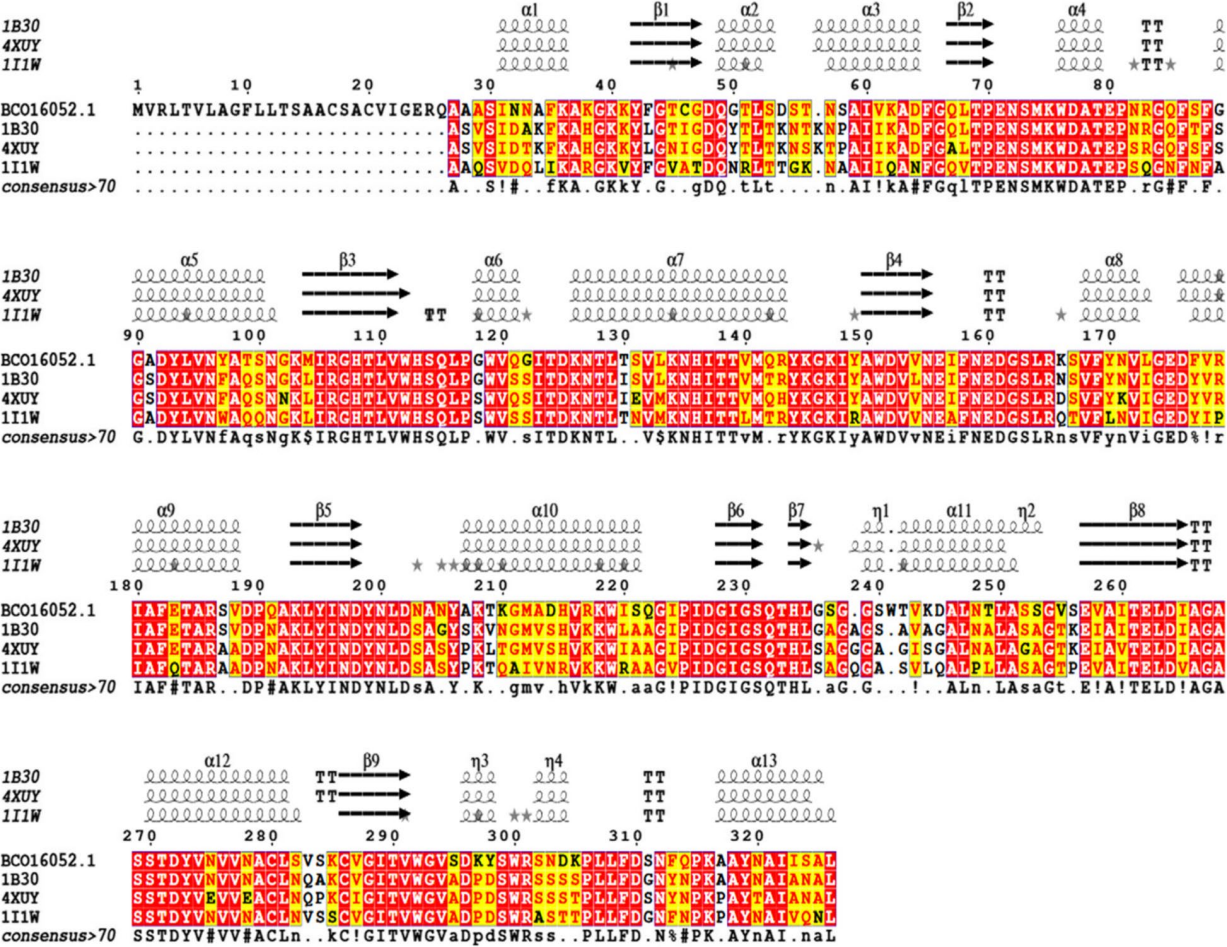
Multiple structure alignment of deduced amino acids sequence of *xynC* gene with *Aspergillus niger* (PDB 4XUY _A) and *Penicillium simplicissimum* DSM 17393 (PDB ID 1B30)

The deduced amino acid sequence of *XynC* (protein ID: BCO16052.1) was aligned against the PDB using BLASTp to conduct a sequence homology search and comparative modeling. Significant sequence similarities were 78% with *Aspergillus niger* xylanase (PDB ID 4XUY) and 73% with *Penicillium simplicissimum* DSM 17393 (PDB ID 1B30). The sequence alignment of xylanase and templates in the ESPript server and secondary structure prediction of the xylanase protein are presented (Fig. 21). According to the characterization of the xylanase model by the DSSP program, the secondary structure of xylanase is composed of 13 𝛼-helixes and 9 𝛽-sheets.

### Homology modeling and validation of xylanase

**Fig. 22:**
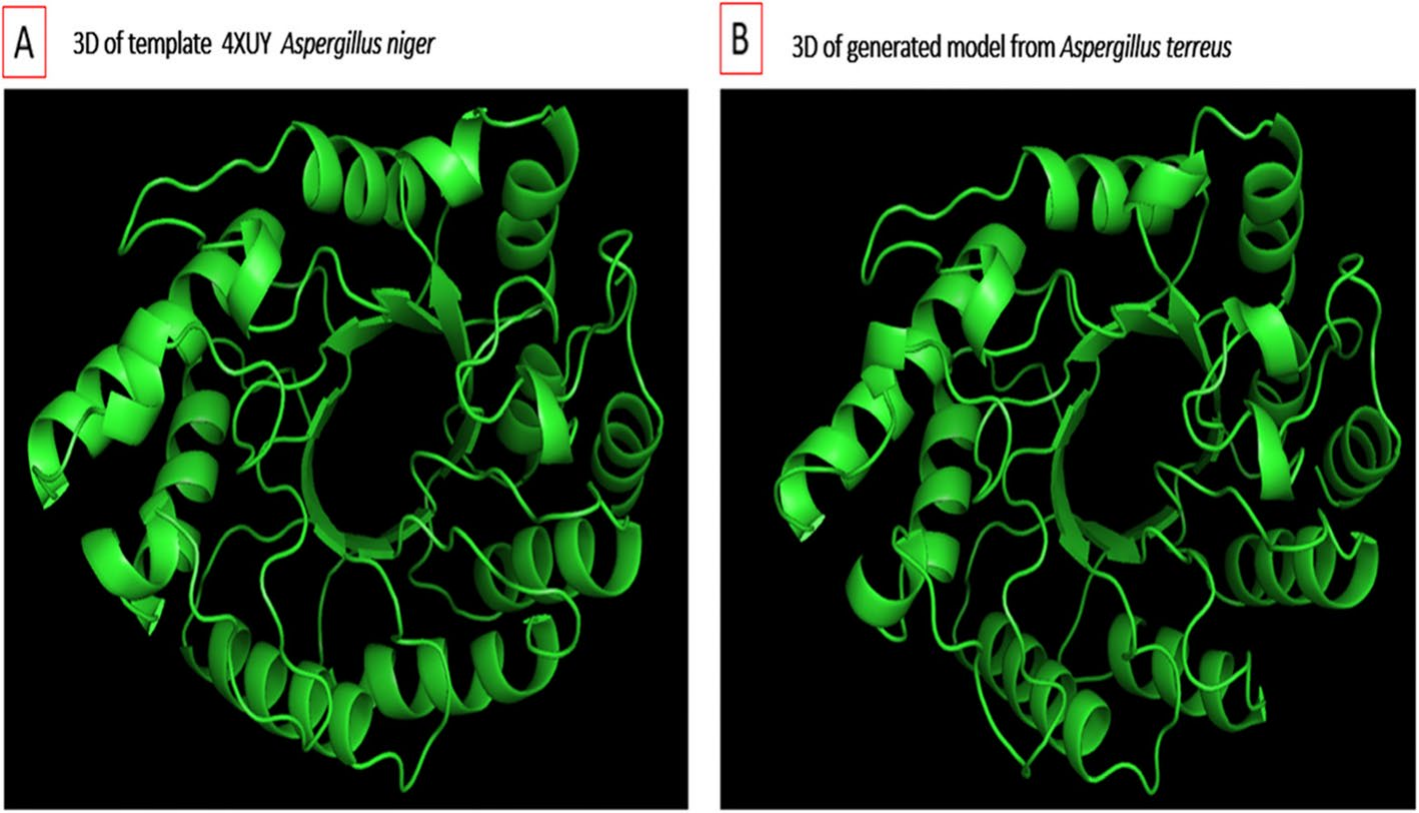
**a** Super imposition of modeled xylanase gene from *A. terreus* (predicted model) and **b** template *Aspergillus niger* (PDB 4XUY _A) through cartoon representation

**Fig. 23:**
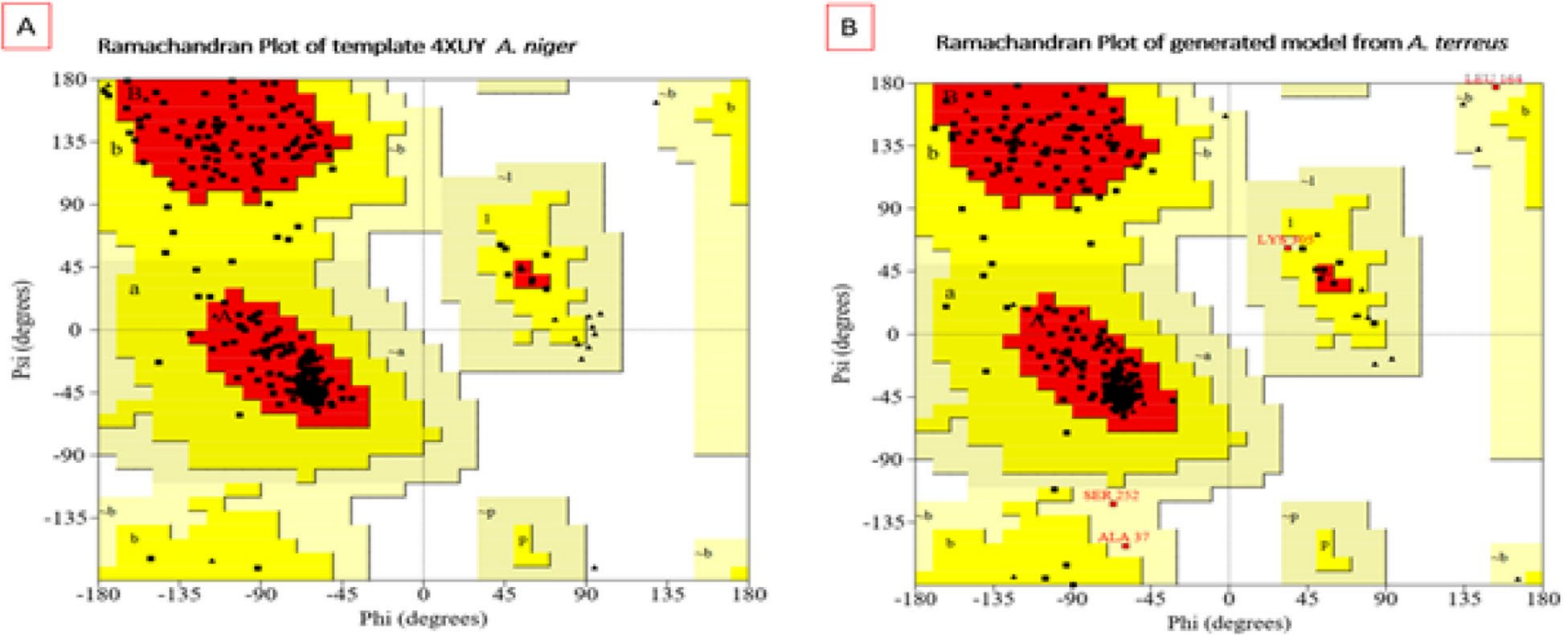
Ramachandran plot for template *Aspergillus niger* (PDB 4XUY _A). **a** Modeled xylanase protein. **b** Obtained PROCHECK

Since sequence similarities were higher with *Aspergillus niger*, X-ray structural coordinate files of xylanase from this strain (PDB 4XUYA, identity 78%) were used to conduct homology modeling and build the 3D structure of xylanase produced from the *A. terreus* strain RGS.Eg-NRC. Then, best hit (*E*-value 0) crystal structures of the xylanase protein were used as templates to construct 3D models of xylanase based on sequence similarity, residues completeness, crystal resolution, and functional similarities. Based on the DOPE score, top-scoring models (Fig. 22) were selected for energy minimization and validation studies out of the 300 predicted xylanase models. Subsequently, the selected model was subjected to energy minimization using YASARA Server force fields of the Swiss-PdbViewer, after which the generated model was assessed using general stereochemical parameters by PROCHECK, VERIFY3D, and ERRAT of the SAVES server. As a result, the Ramachandran plot of the energy-minimized model of xylanase structures was also generated. The x-axis of the Ramachandran plot is split into four quadrants, which include the low-energy region, the allowed region, the generally allowed in region, and the disallowed region (Fig. 23). PROCHECK analysis of the 3D modeled xylanase protein revealed that while 90.2% of residues fell in the most favored regions of the Ramachandran plot, 8.3% fell in the additional allowed regions, 1.5% in the generously allowed regions, and no residue was in the disallowed regions, indicating that the generated model was of good quality. Similarly, template 4XUY of *A. niger* had corresponding values, with 91.3% falling in the most preferred regions, 8.7% in additionally allowed regions, and no residues in the generously allowed and disallowed regions of the Ramachandran plot. Furthermore, using VERIFY3D and ERRAT at the SAVES server, the overall quality factor and compatibility of the atomic model (3D) with amino acid sequence (3D-1D) for the model were observed as 83.02% and 78.94. Thus, the Ramachandran plot, ERRAT, VERIFY3D, and PROSA results confirm that the generated model was reliable and of good quality.

### Docking and molecular interaction studies with the three-dimensional model of xylanase

**Fig. 24:**
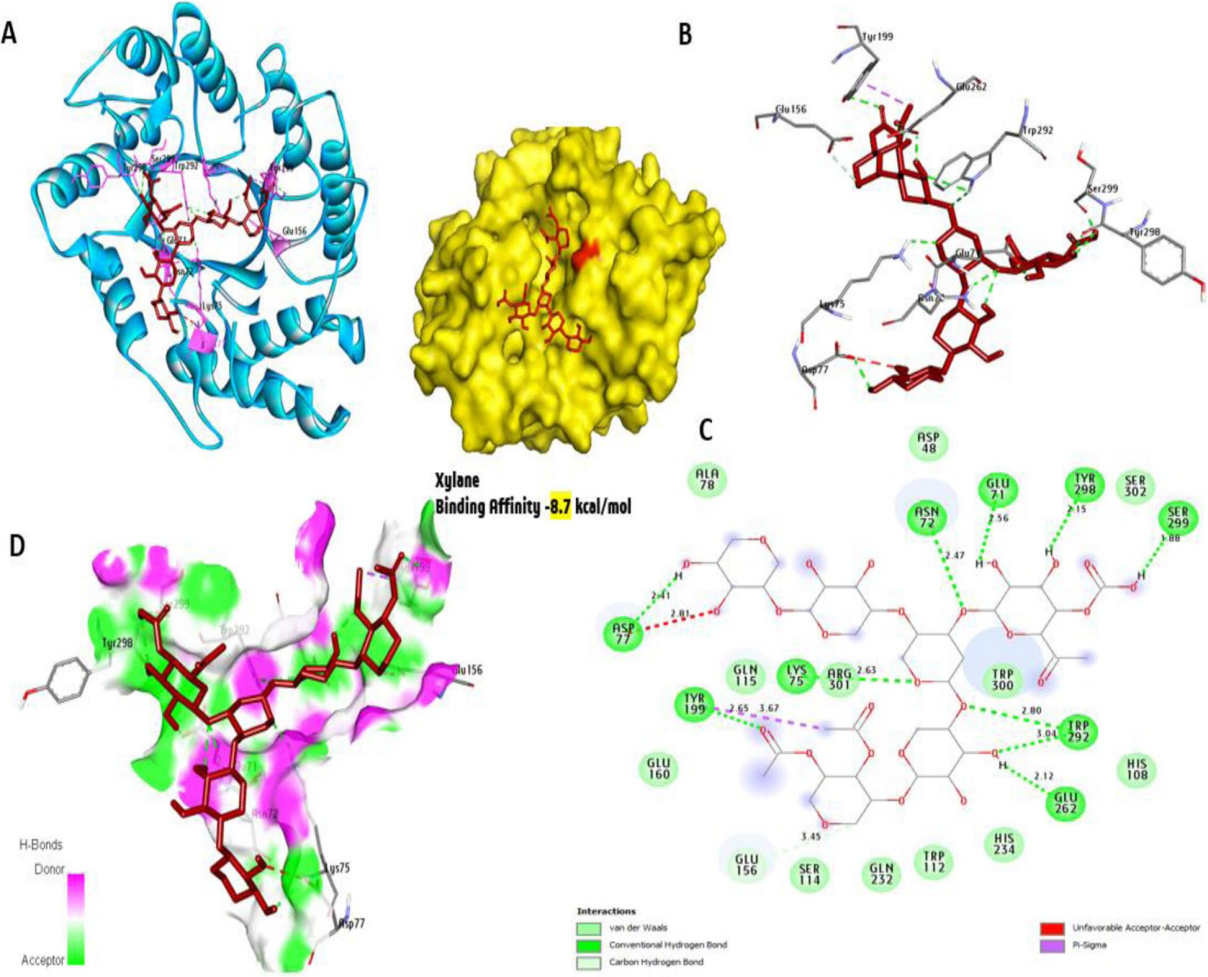
Molecular interactions of xylan with amino acids of the 3D model of xylanase with the best binding mode in the pocket of protein (with ligand as color sticks). **A** Complex interaction with sticks red color, **B** 3D interaction amino acid residues involved in the interaction (with ligand as color sticks), **C** 2D interaction binding interaction of ligands with an amino acid with hydrogen bond (green dash line), and **D** display receptor surface with H-bond donor and acceptor

Figure 24 summarizes the docking results of xylan with the 3D xylanase model. The results showed an interaction affinity with a score of −8.7 kcal/mol that formed 10 hydrogen bonds with Tyr298, Ser299, Tyr199, Glu71, Asn72, Lys75, Asp77, Trp292, and Glu262. Furthermore, while we observed three nonhydrogen bond interactions within the activity pocket, one Pi-sigma bond (with residue Tyr199) and only one carbon-H (with Glu156) were also formed.

## Discussion

Xylanases have several industrial applications, including hydrolysis, lignocellulosic feed stuff nutritive enhancement, wine and liquid clarification, biobleaching of craft soft tissue during paper manufacturing, food additives, poultry, improved handling of coffee doughs, enhanced plant oils, and optimized starch extraction [40, 41]. They can also be used to generate cellulose [3] and during biocontrol [6, 42]. Among all xylanase-producing fungi, since *Aspergillus* sp. is the most common commercial source of xylanase, those with high productivity yields are particularly interesting [6, 42, 43]. Internal transcribed spacer (ITS)-based identification has been considered effective in taxonomy identification because it is accurate and quick and only requires a small amount of sample. In this study, the ITS region of the rRNA encoding gene was successfully employed to identify a potent isolate *Aspergillus* strain: *A. terreus* RGS.Eg-NRC. Many researchers have also used the ITS as a fungal barcoding gene for fungal identification [12]. For example, a previous study [44] employed the ITS gene for the identification of some indigenous *Beauveria* isolates. Moreover, it is efficiently used to identify some mushroom isolates, providing a robust tool for mushroom characterization for human benefits [12]. The production of enzymes was achieved using solid-state fermentation technique, where the xylanase productivity was initially 15.36 U/gds but dramatically increased to 236 U/gds. This effort also involved the fermentation process’s cost-effective and required optimization in enhancing yield. Furthermore, this work used single-factor optimization to facilitate the fermentation process, followed by statistical designs incorporating the effects of various carbon substrates, moistening agents, nitrogen sources, and additives. Based on the differences in substrate accessibility, other substrates sustained diverse but lower amounts of xylan activity than *Ricinus communis*. *Ricinus communis* waste is an efficient, low-cost carbon source that can be detoxified by fermentation to produce various microbial enzymes. Therefore, it has been employed in rapidly manufacturing xylanase [13, 45]. Subsequently, statistical optimization was conducted using PBD, which indicated the enzyme productivity impacting variable. Multiple regression analysis findings later revealed that all understudied factors were significant. In a previous study [46], the model accuracy was confirmed when the R2 value was more than 0.9. Accordingly, with an even higher R2 score of 0.999, the applied model was considered quite accurate in this study, implying that the examined variables caused a variance of 99.9% in enzyme activity. Additionally, the main estimated effects for the analyzed variables revealed that carbon, the moistening agent, and time had the highest main effects of all understudied. As a result, these three factors were subjected to further investigation to confirm their validity. The residual plot revealed that, on average, the model was right for all observed data. Results also showed that the xylanase activity in this study increased after optimization to reach about 12.1-fold, which was, of course, higher than that reported previously [47] using SSF of wheat straw and *A. niger*, whose productivity increased about threefold. Notably, the low cost of *Ricinus communis* has also compelled many researchers interest in its use for producing high-value products besides enzymes such as bioethanol [48]. Thus, investigations showed that the partially purified enzyme was most active at pH 6, where it remained stable for 2 h. This finding agrees with earlier studies, such as those on the chemical characterization of xylanases. Studies reported have also shown that xylanases produced by *Aspergillus* sp. are acidic, with a pH of 4.8 for *A. terreus* [49], 5.0 pH for *A. oryzae* LC1 [50], 5.5 pH for *A. fumigatus* FC2-2 [51], and 6.0 pH for *A. nidulans* [52] and *Aspergillus niger* [47]. Besides, the maximum activity of xylanase was indicated at 55 °C. Nevertheless, considering other xylanases from different species, the xylanase yield in this study was higher than that recorded in other species but at lower temperatures. For example, the maximum activity in *A. niger* was at 50 °C [43], and *Trichoderma inhamatum* was at 50 °C [53]. However, the yield was still similar to that of *Aspergillus oryzae* at 55 °C [54]. Alternatively, because the effective application of end product results from the degradation of *Ricinus communis* by xylanase, resultant XOS mixtures exhibit good antioxidant activities, which increase gradually with an increasing concentration to 1000 μg/ml. For instance, a previous study [55] determined the antioxidant activity of different concentrations of XOS (0.1–4 mg/ml) to be 71% and 78%, respectively, at a concentration of 2 mg/ml. Accordingly, our results agree with yet another study [56], which recorded that the scavenging activity gradually increased constantly to 85.7% for DPPH and 65.5% for hydroxyl radical at a concentration of 2.5 mg/ml. A recent study [57] also announced that the antioxidant activity of XOS may be attributed to the presence of ester-linked hydroxycinnamic acid derivatives, such as ferulic acid, coumaric caffeic acid, syringic acid residues, and methyl-glucuronic acid ramifications on the xylan chain. Moreover, research [58] reported that the antioxidant capacity of XOS obtained from arabinoxylan correlated with the degree of ferulic acid substitution. Subsequently, this study used a PCR method to isolate the *xynC* gene. According to sequence alignment, the xylanase gene encodes 326 amino acids with a molecular weight of 36 kDa. A previous study [11] showed that the full-length xylanase gene (981 bp) from *A. terreus* BCC129 produced a reasonable result. Besides, the strain of *Penicillium citrinum* harbors a xylanase-encoding gene (*xynB*) of 1501 bp in length to encode a protein of 87 amino acids (endo-1,4-beta-xylanase) and has a 264-bp ORF [59]. Xylanase genes have also been identified from various bacteria, including alkaliphilic *Bacillus firmus* [60], *Paenibacillus curdlanolyticus* MP-1 [61], *Streptomyces olivaceoviridis* A1 [62], and *A. niger* BCC14405 [63]. Next, based on in silico simulation using binding affinity and molecular interaction, we discovered that xylan was a promising substrate. A study [64] confirmed our findings, stating that xylanase aids in decomposing linear polysaccharides, such as xylan, subsequently transforming them into xylose. As a result, hemicellulose in plant cell walls is broken down. The substrate selectivity of xylanase from the *A. terreus* strain RGS to preferentially hydrolyze xylan is thus confirmed by our in silico study observation, as previously observed by an earlier empirical study [65, 66].

## Conclusion

High and efficient xylanase production from the *A*. *terreus* strain RGS.Eg-NRC isolated from agro-waste was achieved using caster cake (*Ricinus communis*) as a new and low-cost carbon source by the SSF method. Xylanase activity increased 12.1-fold after optimization to reach (245 U/g). A subsequent cytotoxic activity test then confirmed the safety of xylanase produced from *Ricinus communis*. Based on this study, XOS produced from xylan degradation by xylanase is proposed as a potential antioxidant and antitumor agent.

## Data Availability

The datasets used and/or analyzed during the current study are available from the corresponding author on reasonable request.

## Abbreviations

ITS: Internal transcribed spacer
SSF: Solid-state fermentation
CCD: Central composite design
XOS: Xylooligosaccharides
Ea: Activation energy
EAC: Ehrlich ascites carcinoma
SDS-PAGE: Sodium dodecyl sulfate-polyacrylamide gel electrophoresis
RSM: Responses methodology
GH: Glycosyl hydrolase
GH10: Glycosyl hydrolase families 10
GH11: Glycosyl hydrolase families 11
pI: Isoelectric point
OVAT: One variable at a time
PB: Plackett and Burman
*xyn*C: Xylanase encoding gene
DNSA: 3,5-Dinitrosalicylic acid
PCR: Polymerase chain reaction
DPPH: 2,2-Diphenyl-1-picryl-hydrazyl-hydrate
ABTS: 2, 2′-Azino-bis-3-ethylbenzothiazoline-6-sulfonic acid
BHT: Butylated hydroxytoluene
FRAP: Ferric reducing antioxidant power
EACC: Ehrlich ascites carcinoma cells
FTIR: Fourier transform infrared spectroscopy
PDB: Protein Data Bank
